# Acyltransferases Regulate Oil Quality in *Camelina sativa* Through Both Acyl Donor and Acyl Acceptor Specificities

**DOI:** 10.3389/fpls.2020.01144

**Published:** 2020-08-14

**Authors:** Ida Lager, Simon Jeppson, Anna-Lena Gippert, Ivo Feussner, Sten Stymne, Sofia Marmon

**Affiliations:** ^1^ Department of Plant Breeding, Swedish University of Agricultural Sciences, Alnarp, Sweden; ^2^ Department of Plant Biochemistry, Albrecht-von-Haller Institute for Plant Sciences, University of Goettingen, Goettingen, Germany; ^3^ Göttingen Center for Molecular Biosciences (GZMB), University of Goettingen, Goettingen, Germany

**Keywords:** Camelina, Kennedy pathway, fatty acid composition, acyltransferase, diacylglycerol acyltransferase, diacylglycerol, triacylglycerol, phosphatidylcholine

## Abstract

*Camelina sativa* is an emerging biotechnology oil crop. However, more information is needed regarding its innate lipid enzyme specificities. We have therefore characterized several triacylglycerol (TAG) producing enzymes by measuring *in vitro* substrate specificities using different combinations of acyl-acceptors (diacylglycerol, DAG) and donors. Specifically, *C. sativa* acyl-CoA:diacylglycerol acyltransferase (DGAT) 1 and 2 (which both use acyl-CoA as acyl donor) and phospholipid:diacylglycerol acyltransferase (PDAT, with phosphatidylcoline as acyl donor) were studied. The results show that the DGAT1 and DGAT2 specificities are complementary, with DGAT2 exhibiting a high specificity for acyl acceptors containing only polyunsaturated fatty acids (FAs), whereas DGAT1 prefers acyl donors with saturated and monounsaturated FAs. Furthermore, the combination of substrates that resulted in the highest activity for DGAT2, but very low activity for DGAT1, corresponds to TAG species previously shown to increase in *C. sativa* seeds with downregulated DGAT1. Similarly, the combinations of substrates that gave the highest PDAT1 activity were also those that produce the two TAG species (54:7 and 54:8 TAG) with the highest increase in PDAT overexpressing *C. sativa* seeds. Thus, the *in vitro* data correlate well with the changes in the overall fatty acid profile and TAG species in *C. sativa* seeds with altered DGAT1 and PDAT activity. Additionally, *in vitro* studies of *C. sativa* phosphatidycholine:diacylglycerol cholinephosphotransferase (PDCT), another activity involved in TAG biosynthesis, revealed that PDCT accepts substrates with different desaturation levels. Furthermore, PDCT was unable to use DAG with ricineoleyl groups, and the presence of this substrate also inhibited PDCT from using other DAG-moieties. This gives insights relating to previous *in vivo* studies regarding this enzyme.

## Introduction

*Camelina sativa* (L.) Crantz is an ancient oil crop that has been almost abandoned in modern agriculture. However, during the last decade, it has caught considerable interest in agricultural and biotechnological research. One of the reasons for the growing interest is that it can easily be transformed by the floral dipping method (Lu and Kang, 2008), like the model oil seed plant * Arabidopsis thaliana*. *C. sativa* has the potential to become a new model plant for breeding oil crops. It has 10 times larger seeds than *Arabidopsis*, which facilitates biochemical studies. In contrast to *Arabidopsis*, *C. sativa* has a reasonable yield of both seeds and seed oil per area when grown in the field. New oil qualities engineered in *C. sativa* can therefore be evaluated industrially, as the amount of oil produced is sufficient for product testing (Zubr, 1997; Usher et al., 2017).

Many of the biotechnological efforts in *C. sativa* have been centered toward changing its seed oil composition to fit various uses. Striking examples of what can be done in this area is the development of *C. sativa* seed oil consisting of 85% unusual acetyl-triacylglycerols (acetyl-TAGs), *i.e.*, two long chain acyl groups and one acetyl groups esterified to glycerol (Liu et al., 2015), and unprecedented high levels of the nutritionally valuable very long chain omega-3 fatty acids, eicosapentanoic acid and docosahexanoic acid in the seed oil (Ruiz-Lopez et al., 2014). Despite the great interest and attempts to modify the composition of *C. sativa* oil, only a few studies concerning the regulation of the oil synthesis and composition in the *C. sativa* seeds have been published (Pollard et al., 2015; Marmon et al., 2017) but without characterizing the enzymes involved. This basic knowledge will greatly aid genetic engineering of oil quality in *C. sativa.*

**Figure 1:**
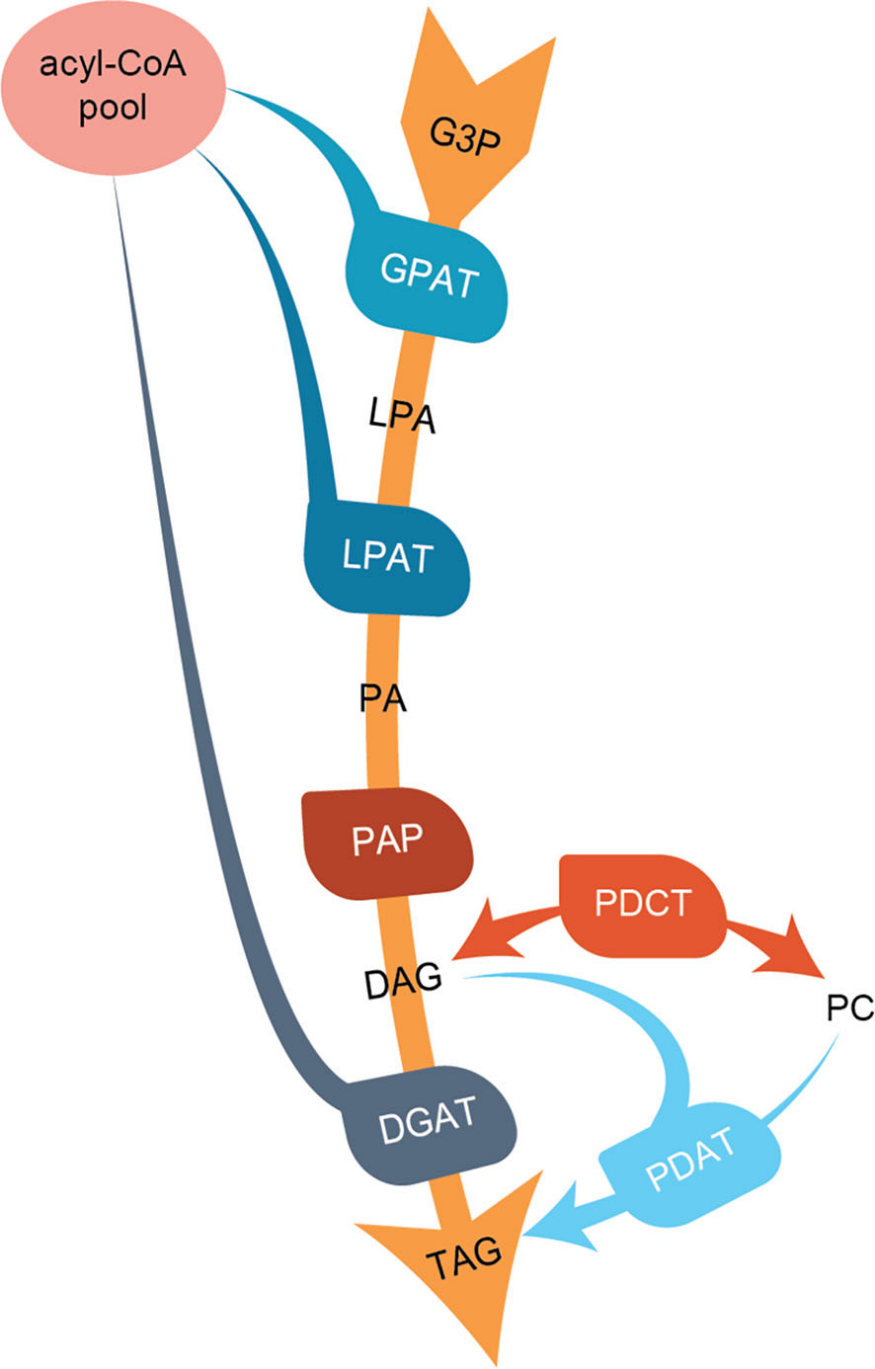
Overview of the *de-novo* triacylglycerol formation in seeds. Abbreviations for lipids (black letters): G3P, glycerol-3-phosphate; LPA, lyso-phosphoatidic acid; PA, phosphatidic acid; DAG, diacylglycerol; PC, phosphatidylcholine; TAG, triacylglycerol. Abbreviations for enzymes (white letters): GPAT, G3P acyltransferase; LPAT, lysophosphatidylacyltransferase; PAP, Phosphatidate phosphatase; PDCT, PC : DAG cholinephosphotransferase; DGAT, acyl-CoA : DAG acyltransferase; PDAT, phospholipid:DAG acyltransferase.

In the present work, we characterize three *C. sativa* types of enzymes involved in regulating TAG composition in seeds; the acyl-CoA:diacylglycerol acyltransferases (DGATs), the phospholipid:diacylglycerol acyltransferase (PDAT), and phosphatidylcholine:diacylglycerol phosphocholine acyltransferase (PDCT). Both DGAT and PDAT catalyze the acylation of diacylglycerols (DAGs), leading to the formation of TAG. DGATs use acyl-CoA as an acyl donor substrate, whereas PDAT uses acyl groups from the *sn*-2 position of phosphatidylcholine (PC) (Ståhl et al., 2004). PDCT interconverts PC and DAG through the transfer of phosphocholine groups from PC to DAG (Lu et al., 2009). An overview of the pathways leading to TAG formation and the studied enzymes is shown in **Figure 1**.

DGATs are represented in plants by at least two enzymes, DGAT1 and DGAT2, which are only distantly related in an evolutionary sense (see Xu et al., 2018, for review). A third enzyme with DGAT activity, DGAT3, has also been identified, although its physiological role in TAG synthesis has not yet been established (Aymé et al., 2018). An *Arabidopsis* DGAT1 mutant has lower levels of seed oil and increased levels of polyunsaturated fatty acids (FAs) (Zou et al., 1999). Downregulation of DGAT1 in *C. sativa* also leads to an increase in α-linolenic acid (18:3) but without significant changes in total seed oil content (Marmon et al., 2017). Knock-out mutations of DGAT2 in *Arabidopsis* had no significant effect on either seed TAG content or its FA composition (Zhang et al., 2009). Expression of foreign DGAT2 variants from species accumulating high amounts of unusual FAs was shown to increase the levels of these particular FAs in the seed TAG of transgenic *Arabidopsis* already harboring the respective genes responsible for the synthesis of the unusual FAs (Burgal et al., 2008; Li et al., 2010).

The characterization of DGAT properties mostly focused on DGAT1 (Lung and Weselake, 2006; Xu et al., 2008; Aznar-Moreno et al., 2015; Xu et al., 2018), whereas DGAT2 was predominantly studied from plants accumulating unusual FAs in their TAG, such as ricinoleic acid (12-hydroxy-octadeca-9*Z*-enoic acid), α-eleostearic acid (octadeca-9*Z*,11*E*,13*E*-trienoic acid), and erucic acid (eicosa-11*Z*-enoic acid) (Shockey et al., 2006; Burgal et al., 2008; Demski et al., 2019; Jeppson et al., 2019)

The majority of DGAT *in vitro* studies only looked at the specificity toward acyl-CoA, with a few reporting on the selectivity toward both the acyl donor and acyl-CoA (Shockey et al., 2006; Xu et al., 2008; Jeppson et al., 2019). Jeppson et al. (2019) showed that DGATs displayed big differences in their specificities toward individual DAG species. The different combinations of acyl-CoA and DAG species influenced the selectivity of the DGATs toward the acyl-CoA moiety; this would have not been revealed if just one acyl acceptor had been used to measure the acyl-CoA specificity.

*Arabidopsis* PDAT knock-out mutants showed no change in either the levels or composition of seed TAG (Ståhl et al., 2004; Mhaske et al., 2005). However, downregulation of PDAT in a DGAT1 *Arabidopsis* mutant background led to drastically decreased TAG levels and abnormal embryo development (Zhang et al., 2009). Downregulation of PDAT activity in *C. sativa* seeds through an artificial microRNA (amiRNA) approach resulted in a slightly changed acyl composition of seed TAG with decreased 18:3, whereas overexpression of PDAT in the same species increased 18:3 levels in TAG substantially (Marmon et al., 2017).

PDAT specificity studies only measured the activity toward different acyl-PC species, but none investigated combinations of different PC and DAG species. Published reports showed that PDAT in *Arabidopsis*, sunflower and safflower prefers 18:2-PC and 18:3-PC over 18:1-PC (Ståhl et al., 2004; Banaś et al., 2013). Further, castor bean and *Crepis palaestina* PDATs were shown to have high specificities for ricinoleoyl-PC and vernoleoyl (12,13-epoxy-octadeca-9*Z*-enoic acid) acyl groups in PC, the main acyl groups in their seed TAG, respectively (Dahlqvist et al., 2000). Overexpression of castor bean PDAT in transgenic *Arabidopsis* expressing the castor bean Δ^12^hydroxylase led to a significant increase in ricinoleic acid in TAG and restored oil content to near wildtype (van Erp et al., 2011).

Mutating or downregulating PDCT substantially decreases the levels of polyunsaturated FAs in seed TAG in *Arabidopsis* and rapeseed (Lu et al., 2009; Bai et al., 2020). A few studies reported a direct *in vitro* assay of recombinant PDCT (Lu et al., 2009; Hu et al., 2012; Bai et al., 2020). However, long before the gene was discovered, *in vitro* enzyme activity had already been demonstrated in assays with microsomal fractions of developing seeds with [^14^C]glycerol labeled glycerol-3-phosphate and acyl-CoA (Stobart and Stymne, 1985).

Our work shows that different combinations of acyl acceptors and acyl donors have great effects on *C. sativa* DGAT and PDAT specificities. The specificities obtained corroborate very well the changes seen in TAG species of *C. sativa* seeds with either silenced DGAT1 or enhanced PDAT activities, resp., (Marmon et al., 2017). Our *C. sativa* PDCT assays allowed us to measure the kinetics of DAG being converted to PC during different rates of *de novo* synthesized DAG. Furthermore, we show that DAG with ricinoleoyl groups is not used by *C. sativa* PDCT and inhibits the transfer of non-hydroxylated DAG into PC.

## Materials and Methods

### Chemicals

Non-radioactive fatty acids were obtained from Larodan (Stockholm Sweden), [^14^C]glycerol labeled glycerol 3-phosphate (G3P), [^14^C]glycerol, [^14^C]-labeled 18:1 and 18:2 fatty acids from Perkin Elmer, and [^14^C]18:3 from American Radiochemicals. CoA, glycerol phosphocholine, *sn*-1,2-18:1-DAG, unlabeled G3P, 16:0-lysophosphatidylcholine, and phospholipase C (from *Clostridium perfringens*) were obtained from Sigma Aldrich. Acyl-CoAs were prepared according to the method devised by Sánchez et al. (1973). *sn*-1-16:0-*sn*-2-[^14^C]acyl-PC was prepared by reacting the *sn*-1-16:0-lysophosphatidylcholine with mixed anhydride of the desired fatty acid according to Kanda and Wells (1981). *sn*-1,2-acyl-DAG was prepared by first reacting the mixed anhydride of the desired fatty acid with glycerol phosphocholine to produce PC, which was then treated with phospholipase C in borate buffer, pH. 7.4, to yield *sn*-1,2 diacylglycerols. DAGs with different acyl groups at the *sn*-1 and *sn*-2 position were synthesized by reacting mixed anhydride of the acyl group aimed for the *sn*-1 position with glycerophosphocholine. Newly formed lysophosphatidylcholine (LPC) was isolated and stored overnight in methanol to allow for acyl migration to the more stable *sn*-1-LPC isomer. The LPC was then acylated with the mixed anhydride of the acyl group to be incorporated at the *sn*-2 position of PC. The newly synthesized PC was then treated with phospholipase C to yield the desired *sn*-1,2-DAG. PC and LPC products were separated on silica 60 TLC plates (Merck) in chloroform:methanol:acetic acid:water (85:15:10:3.5 by vol.) and DAG in heptane:diethyl ether:acetic acid (60:40:1 by vol.). Lipid areas were scraped off after wetting the gel with water, eluted with methanol:chloroform (2:1) and extracted into chloroform by the methods devised by Bligh and Dyer (1959). The specific radioactivities were determined by scintillation counting and gas chromatography of the methyl esters after methylation of the lipids using heptadecanoic acid methylester as internal standard. [^14^C]glycerol labeled *sn*-1,2-*rac*-6:0-DAG was prepared as previously described by Jeppson et al. (2019) by acylation of [^14^C]glycerol with mixed anhydride of 6:0 fatty acids (Sánchez et al., 1973) to 6:0-TAG. TAG was isolated after TLC separation and treated with TAG lipase, resulting products were separated on TLC, and the [^14^C]*sn*-1,2-*rac*-6:0 was isolated and eluted from the gel.

### Gene Isolation and Cloning

*C. sativa* DGAT2, PDCT, and PDCT-like genes were cloned from cDNA made from RNA extracted from developing wildtype *C. sativa* seeds, as described in Marmon et al. (2017). *C. sativa* DGAT1 and PDAT genes were cloned from plasmids containing said genes described in Marmon et al. (2017). Primers with the 20 first amino acids codon optimized for yeast were used (**Supplementary Table 6**) to amplify the genes by PCR using Q5 high fidelity DNA polymerase from North England Biolabs, producing blunt end PCR products. The PCR products were purified by separation on agarose gels followed by Nucleospin gel and PCR Clean up kit from Machery Nagel. These where ligated with pYES2/NT vector from Thermo Fisher Scientific using T4 DNA ligase and transformed into chemically competent *Escherichia coli* XL1 Blue. Isolated plasmids were then further used for transformation of *Saccharomyces cerevisiae* H1246 (Sandager et al., 2002), a yeast strain deficient in TAG synthesis genes.

### Yeast and Plant Microsomal Preparation

*C. sativa* plants with a seed-specific overexpression of PDAT and downregulation of DGAT1 are described by Marmon et al. (2017). PDAT overexpression was done in combination with a very weak DGAT1 downregulation, and the results shown are considered to derive from the strong PDAT overexpression, which was 200 times stronger than compared to wildtype (Marmon et al., 2017). Seeds from the same PDAT overexpressing plants were used for microsomal preparations. The plants downregulating DGAT1 were grown in greenhouse at light 200W/m2 16 h/day, day temp 18 to 24°C night temp 15 to 18°C and RH 60%. Developing seeds at mid-stage of seed development (between 12 and 18 days after flowering) were collected, and the microsomal fraction was obtained as previously described (Bafor et al., 1991).

Microsomal preparations of recombinant yeast H1246 were made as previously described (Lager et al., 2013).

### Enzyme Assays

*C. sativa* DGAT1 and DGAT2 activities were measured with acyl-CoA and [^14^C]di-6;0-DAG or radioactive acyl-CoA and non-radioactive DAG in microsomal fractions of H1246 yeast expressing the respective enzymes according to the method described by Jeppson et al. (2019). DGAT activity varied substantially depending on the type of DGAT enzyme and the chain length of the acyl acceptor used (radioactive di-6:0-DAG or long chain DAG). To ensure that less active substrate combinations were accurately monitored, we adjusted the microsomal protein concentrations and incubation times for the different assays as stated below. This way, enough radioactively labeled TAG was generated per assay while keeping acyl-CoA concentrations in excess. Microsomal protein content was determined using the Pierce BCA Protein assay kit from Thermo Fisher Scientific. Specificity assays with microsomal membranes harboring DGAT1 and [^14^C]di-6:0-DAG were performed with 5 nmol [^14^C]6:0-DAG (dissolved in buffer), 5 nmol of acyl-CoA and microsomal membranes corresponding to 40 µg microsomal protein, 100 µg of BSA (fatty acid free), and 5 mM MgCl2 in 50 mM HEPES buffer, pH 7.2, in a total volume of 100 µl at 30°C for 20 min with shaking (1250 rpm/min). DGAT2 assays with [^14^C]6:0 were carried out with the same incubation mixture, but with 5 µg of microsomal protein under shaking (1250 rpm/min) and an incubation time of 8 min. Incubations were terminated by extracting the lipids into chloroform (Bligh and Dyer, 1959). An aliquot of the chloroform phase was subjected to liquid scintillation counting to determine the total radioactivity in this phase.

DGAT assays with long chain DAGs as acyl acceptor were performed as previously described (Jeppson et al., 2019) by adding 20 nmol DAG dissolved in 4 µl of DMSO under vigorously vortexing to 86 µl of incubation mixture containing 40 µg of microsomal protein in 0.1 M HEPES buffer, pH 7.2, containing 5 mM MgCl2. Reactions were initiated by adding 5 nmol [^14^C]acyl-CoA dissolved in 10 µl of water containing 100 µg of fatty acid–free BSA. Incubations were performed at 30°C with shaking (1250 rpm) for 4 min for DGAT1 assays and 30 min for DGAT2 assays. Assays were terminated by extracting the lipids into chloroform (Bligh and Dyer, 1959).

PDAT activity was measured in microsomal fractions from *C. sativa* seeds overexpressing the PDAT (Marmon et al., 2017) in a modified method of Ståhl et al. (2004) as follows: Microsomal fractions corresponding to 60 µg of microsomal protein were freeze dried for 3 to 5 h. Four nmol of *sn*-1-16:0-*sn*-2[^14^C]acyl-PC and 8 nmol of *sn-*1,2-DAG dissolved in 14 µl of benzene were added to the freeze dried microsomal preparation, and the benzene was rapidly removed by a stream of nitrogen after which 100 µl of 0.1M phosphate buffer, pH 7.2, were added. Incubations were allowed to proceed at 30°C with shaking at 1250 rpm for 10 min. The assays were terminated by extracting the lipids into chloroform according to Bligh and Dyer (1959). The formation of [^14^C]TAG from [^14^C]18:2-PC and 18:2-DAG was essentially linear up to 20 min (**Supplementary Figure 1**). For determination of substrate specificities, incubation time was 10 min.

*C. sativa* PDCT was measured in microsomal fractions from H1246 yeast expressing the enzyme. Each assay contained microsomal fractions corresponding to 100 µg of microsomal protein, 12.5 nmol of [^14^C]G3P and 25 nmol of acyl-CoA in 0.1M tris-HCl buffer, pH 7.2 containing 5 mM MgCl2 and 100 µg of BSA in a total volume of 50 µl and incubated at 30°C at times indicated in the figures. The incubation was terminated by extracting the lipids into chloroform phase according to Bligh and Dyer (1959).

### ^14^C-Lipid Analyses

An aliquot of the chloroform phase from the DGATs assay with [^14^C]6:0-DAG was subjected to liquid scintillation counting to determine the total radioactivity in this phase. The remaining chloroform phase was evaporated, the lipids were dissolved in heptane, and the relative amounts of radioactivity residing in DAG and TAG were determined by Raytest Ramona radioactive detector coupled to a HPLC system as previously described (Jeppson et al., 2019).

In DGAT assays of activity with long-chain DAGs and in PDCT assays, the radioactivity of an aliquot of the chloroform phase was used for liquid scintillation counting to determine the total radioactivity in this phase. The remaining of the chloroform phases from the DGAT and PDAT assays were applied to silica 60 TLC plated (Merck) and developed in heptane:diethyl ether:acetic acid (70:30:1 by vol.), and relative proportion of radioactivity resided in TAG was determined by Instant Imager (Packard) electronic autoradiography. In PDCT assays, the remaining chloroform phase was likewise applied to TLC plates and first developed in chloroform:methanol:acetic acid:water (85:15:10:3.5, by vol.) to half the height of the plate. After drying, the plate was re-developed to full length in heptane:diethyl ether:acetic acid (70:50:1), and the relative proportions of the radioactivity in phosphatidic acid (PA), PC, and DAG with no, one and two ricinoleoyl groups were determined by electronic autoradiography.

### Statistics

All data are expressed as means ± SD on triplicate assays. Statistical analysis was performed using OriginPro 2016. The statistical tests used were one-way ANOVA followed by Tukey’s test for comparisons of means. Data were considered statistically significant at p ≤ 0,05. Brown-Forsythe Test was used to determine homogeneity of variance. In all cases the population variances were not significantly different.

## Results

### *C. sativa* DGAT1 and DGAT2 Substrate Specificities

**Figure 2:**
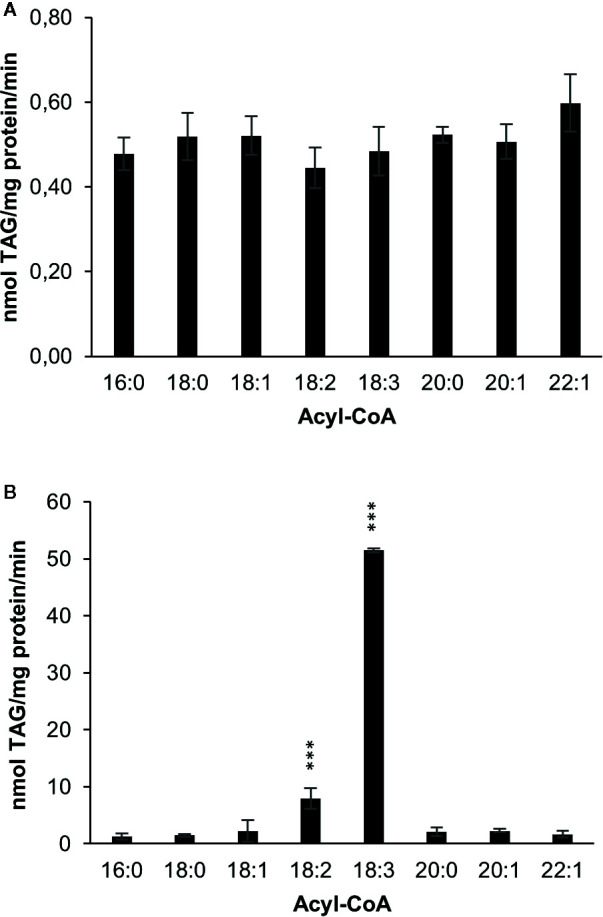
Acyl-CoA specificities of *C. sativa* DGAT enzymes in microsomal preparations from yeast expressing the enzymes, when fed [^14^C]glycerol labeled di-6:0-DAG and different acyl-CoAs. **(A)** DGAT1 and **(B)** DGAT2. Average value shown ± SD, n=3 replicates. Stars over individual bars indicate significant differences according to one-way ANOVA followed by Tukey’s test. ***p ≤ 0.001.

amiRNA – mediated silencing of DGAT1 in *C. sativa* leads to a substantial increase in α-linolenic acid in *C. sativa* seeds (Marmon et al., 2017). In order to investigate whether this change is consistent with a reduced role for DGAT1 and increased role for DGAT2, we measured DGAT1 and DGAT2 substrate specificities after expressing them in the yeast strain H1246 which lacks the capacity to synthesize TAG (Sandager et al., 2002). First we measured acyl-CoA specificities by acylation of di-6:0-[^14^C]glycerol-DAG, an artificial acyl acceptor in microsomal preparations harboring either *C. sativa* DGAT1 or DGAT2 (**Figure 2**). DGAT1 showed no significant differences in activity toward eight different acyl-CoAs tested (**Figure 2A**), whereas DGAT2 showed a strong preference toward 18:3-CoA, which was seven times higher than for 18:2-CoA, the second most preferred substrate. For all other investigated acyl-CoA substrates, TAG synthesis rates of reached less than 5% of the rate measured for 18:3-CoA by DGAT2.

**Figure 3:**
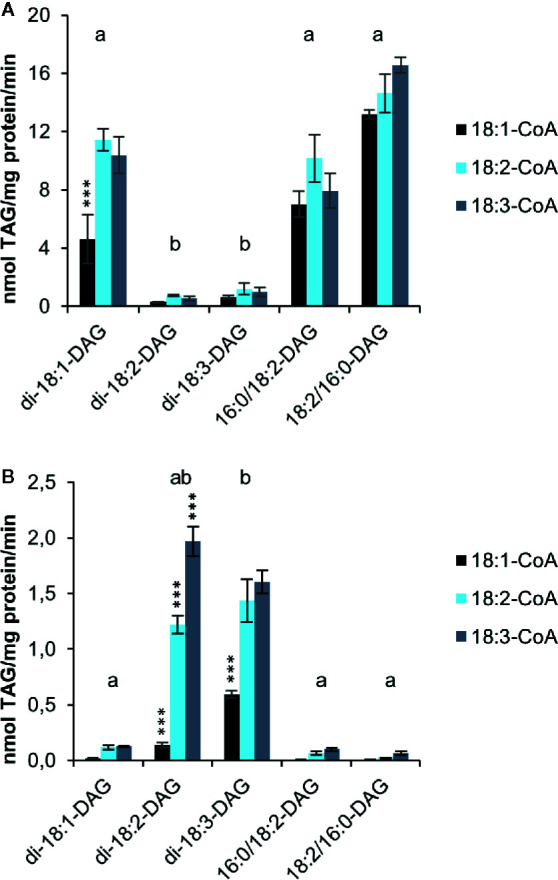
Acyl-CoA and DAG specificities of *C. sativa* DGAT enzymes in in microsomal preparations from yeast expressing the enzymes, when fed exogenous long-chain DAGs and [^14^C]acyl-CoA as indicated in the figure. Background activity (no DAG added) was subtracted in the figure (see **Supplementary Table 1**). **(A)** DGAT1 and **(B)** DGAT2. Average value shown ± SD, n = 3 replicates. Stars over individual bars indicate significant differences compared to the other Acyl-CoA species with the same DAG according to one-way ANOVA followed by Tukey’s test performed on the whole dataset. ***p ≤ 0.001. Different letters over DAG species indicate significant differences between DAG species of all Acyl-CoA combinations at the level of p ≤ 0.001. Complete results of the statistical analysis can be found in **Supplementary Tables 2** (DGAT1) and **3** (DGAT2).

Although the artificial acyl acceptor 6:0-DAG has been used in a number of studies (Banaś et al., 2013; Furmanek et al., 2014; Demski et al., 2019), recent studies showed that the activities of DGATs from *Crambe hispanica* toward different acyl-CoA species are quite different with 6:0-DAG as acyl acceptor than compared to assays using exogenous long-chain DAGs (Jeppson et al., 2019). Moreover, the use of an artificial acyl acceptor does not provide any information about DGAT specificities toward naturally occurring DAGs nor the combinatory effect of different acyl-donor and acyl-acceptor molecular-species. We therefore performed microsomal DGAT assays with combinations of different long-chain DAGs (added in DMSO) and 18:1-CoA, 18:2-CoA or 18:3-CoA (**Figure 3**). The results reveal a striking impact of DAG molecular species on the activities of both DGAT1 and DGAT2. Di-18:1-DAG and *sn*-1-16:0-*sn*-2-18:2-DAG were good acyl acceptors for DGAT1with all acyl-CoAs tested (**Figure 3A**), whereas the reverse was seen for DGAT2 (**Figure 3B**). For those DAG species that were good acyl-acceptors, 18:1-CoA gave lower activities than 18:2-CoA and 18:3-CoA, and this was particularly pronounced for DGAT2. The exceptionally high specificity for 18:3-CoA seen with the di-6:0-DAG acceptor by DGAT2 was not observed with long-chain DAGs; 18:2-CoA was used at similar rates as 18:3-CoA with di-18:3-DAG and about 60% of the rate of 18:3-CoA with di-18:2-DAG. It is noteworthy that *sn*-1-16:0-*sn*-2-18:2-DAG was well accepted by DGAT1 despite the fact that di-18:2-DAG was a very poor acyl acceptor for this enzyme. DGAT2 hardly used this substrate, whereas di-18:2-DAG was well utilized. We also tested *sn*-1-18:2-*sn*-2-16:0-DAG as substrate, a molecular species that is unlikely to occur naturally in significant amounts in *C. sativa*. Extraplastidial long chain lipids are very low in saturated fatty acids at their *sn*-2 position due to lysophosphatidic acid acyltransferases strongly selecting for unsaturated fatty acids over saturated (Li-Beisson et al., 2013). The results were similar to those achieved with the natural positional distribution and DGAT1 displayed an even somewhat better activity with this ‘unnatural’ DAG species.

The specific activities of the enzymes toward long-chain DAGs differed drastically from those with di-6:0-DAG. When DGAT1 was supplied with the best substrate combination of long-chain DAGs and acyl-CoA it showed an over 30 times higher specific activity compared to di-6:0-DAG assays., In contrast, DGAT2 had 25 times lower activity with its best used Acyl-CoA (18:3) when using long-chain di-18:2-DAG compared to di-6:0-DAG. Comparisons in activity between DGAT1 and DGAT2 cannot be made, as the expression levels of the two different enzymes may differ.

DGAT1 control assays showed a measurable background activity, *i.e.*, assays without addition of DAG (only DMSO), whereas there was no detectable background activity in DGAT2 assays (**Supplementary Table S1**).

### Substrate Specificities of *C. sativa* PDAT

**Figure 4:**
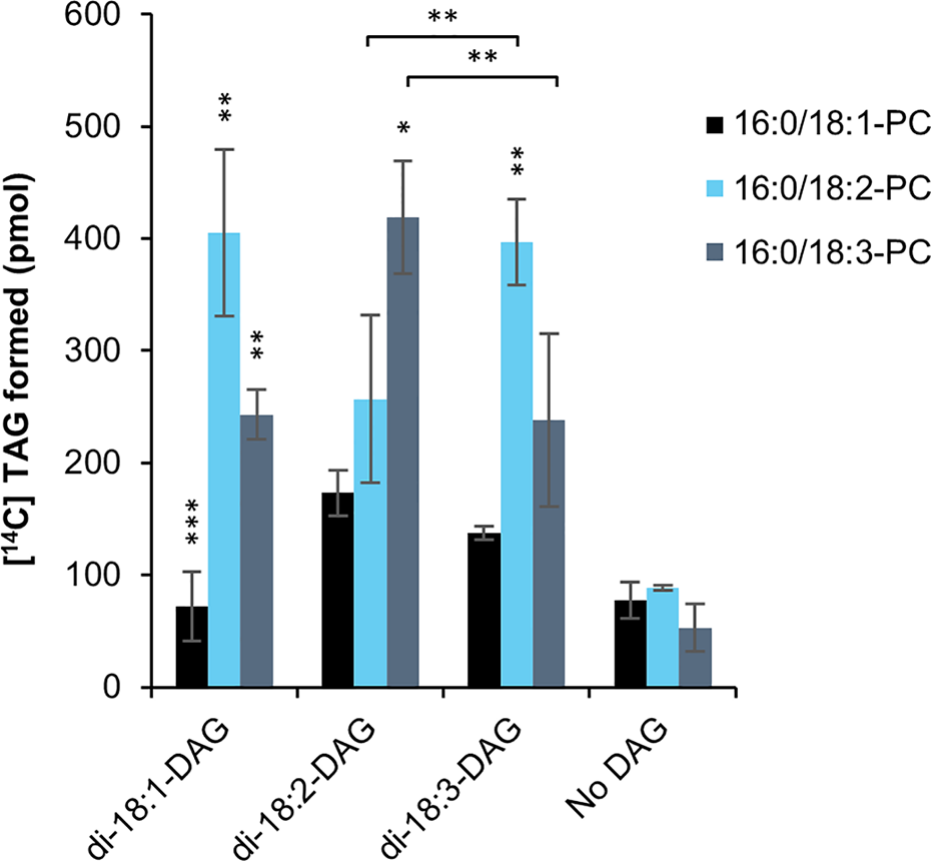
Substrate specificities of *C. sativa* PDAT in microsomal preparations of from developing seeds overexpressing the enzyme, when fed [^14^C]radiolabeled PC and different DAG. Stars over individual bars indicate significant differences compared to the other PC species with the same DAG according to one-way ANOVA followed by Tukey’s test performed on the whole dataset. *p ≤ 0.05, **p ≤ 0.01, ***p ≤ 0.001. Stars at lines between bars indicate significant differences between selected PC and DAG species further discussed. Complete results of the statistical analysis can be found in **Supplementary Table 4**. Average value shown ± SD, n = 3 replicates.

Overexpression of PDAT in *C. sativa* led to a significant increase in 18:2 in the seed oil (Marmon et al., 2017). In order to find a biochemical explanation for these changes, we investigated the substrate specificities of PDAT in microsomal preparations from developing *C. sativa* seeds overexpressing the PDAT. There have been only a few *in vitro* specificity studies of PDAT (Ståhl et al., 2004; Banaś et al., 2013) and all of them only looked at the acyl specificity toward the PC substrate and not at the effect of the acyl composition of the DAG species. We therefore tested the effect of di-18:1-DAG, di-18:2-DAG or di-18:3-DAG on *C. sativa* PDAT activity, using *sn*-1-16:0-*sn*-2-[^14^C]acyl substrate (PC) containing either radioactive 18:1, 18:2 or 18:3 (**Figure 4**), as the dominating acyl species in the *sn*-2 position (Marmon et al., 2017). The background activity did not differ significantly between the different [^14^C]acyl-PC species, indicating a limiting amount of endogenous DAG for acylation. Addition of DAG increased the activity with 18:2-PC and 18:3-PC about 4.5 times compared to background, whereas no significant increase was seen by adding di-18:1-DAG to assays with 18:1-PC (**Figure 4**). Use of 18:1-PC was also significantly lower than that of 18:2-PC and 18:3-PC with the other DAG species added. With di-18:3-DAG added, 18:3-PC gave significantly higher activity than with 18:2-PC, and the reverse was seen when di-18:3-DAG was used (**Figure 4**). Thus, unexpectedly, the acyl acceptor molecular species influenced the specific activity of acyl donor species differently. With di-18:1-DAG, the enzyme preferred 18:2-PC over 18:3-PC, like with di-18:2-DAG. It should be pointed out that whereas di-18:2-DAG and di-18:3-DAG are major DAG species in *C. sativa* seeds, di-18:1-DAG is a very minor species during the stage of rapid seed oil accumulation (Marmon et al., 2017). Comprehensive statistical analyses of significance between all assayed combinations of acyl-CoAs and DAGs are given in **Supplementary Table 4**.

### Characterization of *C. sativa* PDCT Properties

**Figure 5:**
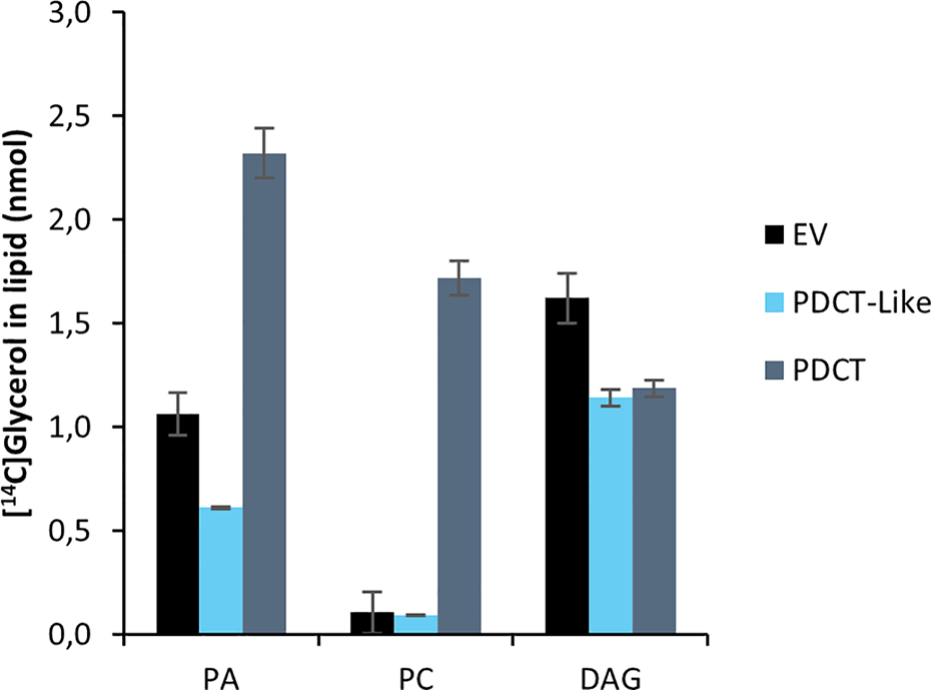
Incorporation of radioactivity into chloroform soluble lipids in microsomal fractions of yeast expressing in the in figure indicated *C. sativa* genes fed [^14^C]glycerol labeled G3P and 18:3-CoA. Incubation time was 60 min. PA, phosphatidic acid; PC, phosphatidylcholine; DAG, diacylglycerol; EV, Empty vector (control); PDCT, PC : DAG cholinephophotransferase. Average value shown ± SD, n = 3 replicates.

PDCT catalyzes the interconversion between DAG and PC through the transfer of the phosphocholine group from PC to DAG. Previously published experiments with microsomal preparations from developing seeds of *C. sativa* incubated with [^14^C]glycerol labeled G3P and acyl-CoA showed a high flux of radioactive glycerol into PC (Marmon et al., 2017), indicating an active PDCT in these seeds. In addition to a PDCT gene, *C. sativa* also harbors a homolog, a PDCT-like protein (ortholog to AT3G15830) with unknown function. We assayed the *C. sativa* PDCT enzyme as well as the PDCT-like enzyme in microsomal fractions of the H1246 yeast strain expressing these genes by incubating the membranes with [^14^C]- G3P and acyl-CoAs. Since H1246 lacks the ability to form TAG, the glycerol backbone of DAG from [^14^C]glycerol-G3P and acyl-CoA, formed *via* the Kennedy Pathway, should enter PC if PDCT is active (see **Figure 1**). Incubation of [^14^C]G3P with 18:3-CoA for 60 min yielded three radioactive lipid species in the membranes expressing the PDCT gene, PA, DAG, and PC with a ratio between DAG and PC of about 0.7:1. The formation of PC and DAG accounted for about 60% of the total activity of the chloroform soluble lipids (**Figure 5**). In membranes expressing either empty vector (EV) or the PDCT-like gene, a similar and very small, yet significant amount of radioactivity was seen in PC, with the majority of the radioactivity observed in DAG (**Figure 5**). The results confirm that the *C. sativa* gene encodes a PDCT, whereas the PDCT-like gene lacks this activity. Since no PDCT homolog is found in yeast, the small amounts of radioactive PC seen both in yeast membranes harboring empty vector and expressing the PDCT-like gene might have been produced by another, unknown, yeast enzyme.

**Figure 6:**
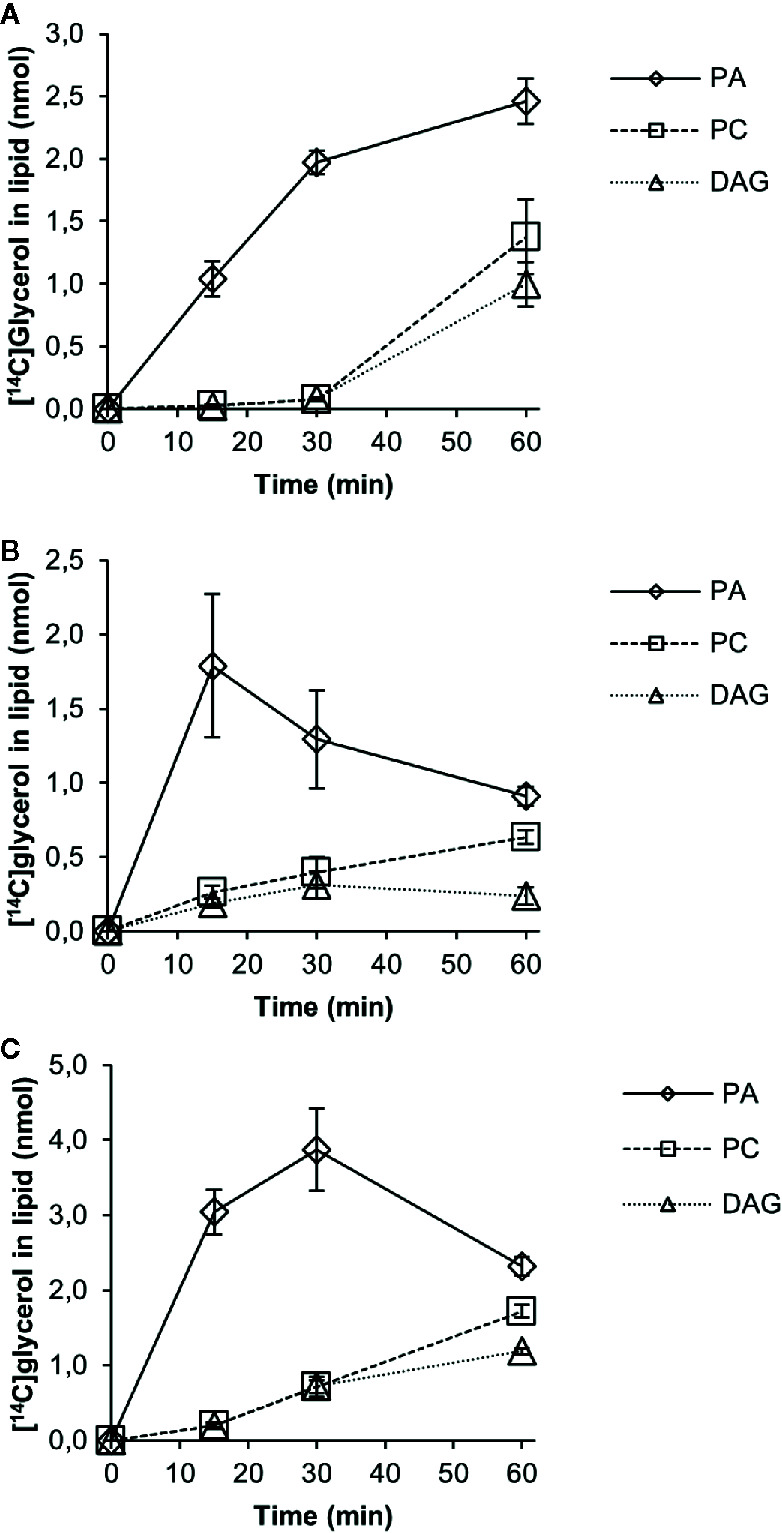
Time-course incorporation of radioactivity into chloroform soluble lipids in microsomal fractions of yeast expressing *C. sativa* PDCT fed [^14^C]glycerol labeled glycerol 3-phosphate and different acyl-CoAs. **(A)** 18:1-CoA, **(B)** 18:2-CoA, and **(C)** 18:3-CoA. PA, phosphatidic acid; PC, phosphatidyl-choline; DAG, diacylglycerol. Average value shown ± SD, n = 3 replicates.

We then followed the [^14^C]G3P derived radiolabel of glycerol in a time-course experiment with 18:1-CoA, 18:2-CoA, or 18:3-CoA in microsomal membranes expressing the PDCT (**Figure 6**). No significant amount of radioactive lysophosphatidic acid (LPA) was detected, and total activity found in chloroform soluble lipids differed substantially between assays with different acyl-CoAs. This indicates that the activity of the first acylation step of G3P (see **Figure 1**), the yeast acyl-CoA:glycerol-3-phosphate acyltransferase (GPAT), is rate-limiting in the production of PA and affected by the acyl-CoA provided. The further conversion of PA to DAG varied depending on the acyl-CoA used in the assays. Assays with 18:1-CoA (**Figure 6A**) showed a clear lag phase during the first 30 min and with very little radioactive DAG or PC accumulating during that period. In assays with 18:1-CoA and 18:3-CoA (**Figures 6A and C**, respectively), the radiolabel of PC and DAG increased in a similar fashion from 30 to 60 min with PC being the major labeled species. However, with 18:2-CoA (**Figure 6B**), the increase of label between 30 and 60 min occurred exclusively in PC, whereas the radioactive DAG level remained more or less constant during that period.

**Figure 7:**
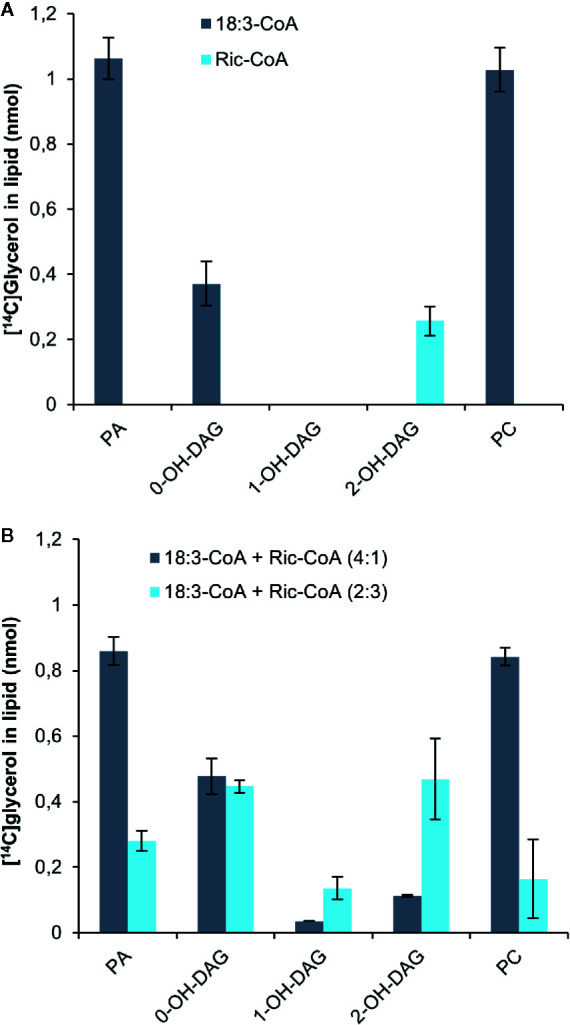
Incorporation of radioactivity into chloroform soluble lipids in microsomal fractions of yeast expressing *C. sativa* PDCT fed [^14^C]glycerol labeled G3P and 18:3-CoA and or ricinoleoyl-CoA. **(A)** 18:3-CoA and ricinoleoyl-CoA (Ric-CoA) as indicated in the figure. **(B)** Mixtures of 18:3 and Ric-CoA in proportions indicated in the figure. Incubation time was 60 min. PA, phosphatidic acid; PC, phosphatidyl-choline; DAG, diacylglycerol. Average value shown ± SD, n = 3 replicates.

Bates and Browse noted from *in vivo* radiolabeling studies that developing *Arabidopsis* seeds expressing the castor bean Δ^12^hydroxylase (which hydroxylates oleic acid to ricinoleic acid) had a 70% reduction in the flow of DAG into PC compared to control plants (Bates and Browse, 2011). They attributed this to the presence of ricinoleoyl groups in DAG inhibiting the conversion of DAG into PC. We therefore incubated yeast H1246 microsomes expressing the *C. sativa* PDCT with [^14^C]G3P and ricinoleoyl-CoA for 60 min and analyzed the radioactivity in the formed lipids. Only a small amount of radioactivity was transferred to the chloroform phase compared to assays with 18:3-CoA (**Figure 7A**). All radioactivity resided in DAG carrying two ricinoleoyl groups (2-OH-DAG), whereas the incubation with 18:3-CoA contained three times more radiolabeled PC than DAG. Furthermore, we incubated the microsomal membranes with [^14^C]G3P and a mixture of 18:3-CoA and ricinoleoyl in 4:1 and 2:3 ratios while keeping the total acyl-CoA concentration the same (**Figure 7B**). The acyl-CoA ratio of 4:1 slightly inhibited the total 14C incorporation into lipids compared to only 18:3-CoA. DAG with one and two ricinoleoyl groups (1-OH-DAG and 2-OH-DAG, respectively) made up 3% and 10% of total DAG + PC radioactivity in these assays. The ratio of radioactivity in PC to that of DAG with no ricinoleoyl groups (0-OH-DAG) decreased from 2.9 with only 18:3-CoA to 1.76. In incubations with 18:3-CoA and ricinoleoyl-CoA, the ratio was 2:3 and less radioactivity was found in the chloroform soluble lipids than in the assays with a ratio of 4:1, and about equal amounts of 0-OH-DAG and 2-OH-DAG were produced, with one third of these levels seen in 1-OH-DAG. The ratio of PC to 0-OH-DAG further decreased to 0.25, a more than 10-fold decrease compared to the ratio with only 18:3-CoA. Thus, we conclude that PDCT cannot use DAG with ricinoleoyl groups, and moreover, the presence of such DAG species also inhibited the transfer of DAG with no ricinoleoyl groups, in this case di-18:3-DAG, to PC.

## Discussion

The aim of this work was to characterize the TAG forming network represented by a number of key enzymes, DGATs, PDAT and PDCT, from C. *sativa*. They are involved in regulating the FA composition of seed TAG, and we aimed to relate the findings to the previously described effects on the FA profile by downregulating DGAT1 and overexpression of PDAT in seeds of *C. sativa* (Marmon et al., 2017). The study revealed strong effects on the substrate specificities of DGAT and PDAT depending on the different combinations of acyl donor and acyl acceptor molecular species. There is no previous report regarding PDAT properties in this respect. We further investigated some properties of the *C. sativa* PDCT, an enzyme for which an *in vitro* characterization has yet been lacking.

### *C. sativa* DGAT1 and DGAT2 Show Different and Complementary Substrate Specificities

The acyl-CoA specificities of *C. sativa* DGAT1 and DGAT2 using di-6:0-DAG as an artificial acyl acceptor were determined in microsomal fractions of yeast expressing these enzymes. DGAT1 showed similar activities with all tested acyl-CoAs, whereas DGAT2 showed a very strong preference for 18:3-CoA. Similar differences in acyl-CoA specificities between DGAT1 and DGAT2 using di-6:0-DAG were previously reported by Demski et al. (2019) for the rapeseed DGAT1 and DGAT2 enzymes, whereas Jeppson et al. (2019) showed that the *Crambe hispanica* subs. *abyssinica* DGAT2 isoforms utilized well a range of different acyl-CoA with this acyl acceptor.

Although we can conclude from our data that DGAT1 and DGAT2 clearly differ in their acyl-CoA specificities using di-6:0-DAG acceptor (**Figure 2**), serious doubts regarding the physiological relevance of these specificities become apparent when performing DGAT assays with different naturally occurring long-chain DAGs as acyl acceptors. These assays showed that DGAT1 and DGAT2 activities had complementary specificities (**Figure 3**). Di-18:1-DAG and 16:0-18:2-DAG were well utilized by DGAT1, whereas the polyunsaturated di-18:2 and di-18:3-DAG were very poor acceptors (**Figure 3A**). DGAT2 however showed the converse activity with good activities with the two polyunsaturated DAG species and very poor activity with di-18:1-DAG and 16:0-18:2-DAG. DGAT1 utilized 18:1-CoA, 18:2-CoA, and 18:3-CoA at similar rates with 16:0-18:2-DAG as acyl acceptor but used 18:1-CoA to a lesser extent when di-18:1-DAG was presented as acyl acceptor. DGAT2 had a clear preference for 18:3-CoA when di-18:2-DAG was acyl acceptor. 18:1-CoA was a poor substrate for DGAT2 with both di-18:2-DAG and di-18:3-DAG (**Figure 3B**). The inconsistencies regarding the acyl-CoA specificity between assays with di-6:0-DAG compared to assays with long-chain DAGs in our study may also be true for other DGAT assays involving artificial acyl acceptors, such as fluorescent analogues (McFie and Stone, 2011; Sanderson and Venable, 2012).

The downregulation of DGAT1 by amiRNA in *C. sativa* seeds led to a substantial increase in the percentage of 18:3 and a decrease in 18:1 and 18:2 in seed TAG (Marmon et al., 2017). It can be speculated that DGAT2 may play a proportionally greater role than DGAT1 in regulating TAG composition in these transgenic seeds than in wildtype. When looking at the relative abundance of different TAG species in these transgenic seeds, a 2.5-fold increase in 18:3/18:3/18:3 (54:9) species was the most prominent difference (Marmon et al., 2017). We here show that DGAT2 works well with 18:3-CoA and di-18:3-DAG, whereas the same substrate combination is utilized very poorly by DGAT1. Further, di-18:3-DAG species dominate (about twofold) over di-18:2-DAG species in *C. sativa* seeds during the developmental stage of active TAG deposition (18 days after flowering) (Marmon et al., 2017).

Thus, the changes in FA composition and molecular species indeed support an enhanced DGAT2 activity compared to DGAT1. The second-largest increase in TAG molecular species seen in the ami-DGAT1 seeds was 16:0/18:3/18:3 (52:6) (Marmon et al., 2017). We did not test 16:0-CoA as an acyl donor in the DGAT assays, but since DGAT2 was nearly inactive with 16:0-18:2-DAG, it is likely that the increase in this particular species is a result of DGAT2 acylation of di-18:3-DAG with 16:0-CoA. tri-18:3-TAG species is rather abundant also in wildtype seeds (Marmon et al., 2017). Considering the negligible activity of DGAT1 in producing this TAG species, it is likely that DGAT2 also contributes to regulating the TAG composition in wildtype. This would then contrast with *Arabidopsis* seeds, where no fatty acid changes in seed TAG was seen in plants mutated in the DGAT2 gene (Zhang et al., 2009).

We also tried to measure the relative proportion between DGAT1 and DGAT2 activity in microsomal preparations from wild type *C. sativa* seeds and ami-DGAT1 downregulated seeds. We did that by combining radioactive di-18:1-DAG or di-18:2-DAG with 18:1-CoA and 18:3-CoA, respectively, thereby presenting the best and worst substrate combinations for both types of DGAT. However, no conclusive data could be obtained due to a high background activity of PDAT (results not shown). Although unlikely, we can therefore not totally exclude that some other (unidentified) enzyme(s) producing TAG with similar properties as the DGAT2 is/are compensating for the reduced DGAT1 activity in the transgenic *C. sativa*.

Naturally occurring DAG species in plants virtually lack 16:0 fatty acids in position *sn*-2 (Li-Beisson et al., 2013). Changing the sn positions of *sn*-1-16:0-*sn*-2-18:2 of DAG to *sn*-1-18:2-*sn*-2-16:0 somewhat increased DGAT1 activity compared to the naturally occurring positional isomer and slightly changed the acyl-CoA specificity profile. DGAT2 showed minute activity with both stereo-isomers (**Figure 3**). The results raise interesting questions regarding the nature of the binding site of the two acyl components of DAG, indicating that the enzyme cannot distinguish the *sn*-1 from the *sn*-2 position, perhaps due to a shared binding site for both acyl groups. A more detailed study of DGAT activities with various positional isomers of DAG species is likely to give more information regarding the positional effects of the different acyl groups in DAG on DGAT activities. The stereo-isomer sensitivity may differ between DGAT1 and DGAT2 isoforms as these are probably the result of convergent evolution since they share little/no sequence homology (Turchetto-Zolet et al., 2011).

The DGAT1 background activity, *i.e.*, assays without exogenous DAG added in yeast H1246 microsomal preparations expressing the DGATs, was measurable with DGAT1 but below detection limit with DGAT2 (**Supplementary Table 1**). This is in line with the report of Jeppson et al. (2019) for Crambe DGATs, which showed that endogenous yeast DAG was a very poor acyl acceptor for Crambe DGAT2, even when supplied exogenously in amounts corresponding to the other added DAG species. Endogenous microsomal yeast DAG is composed of di-monounsaturated and saturated-monounsaturated species, and *C. sativa* DGAT2 showed very poor activity with di-18:1-DAG and 16:0-18:2-DAG. Thus, differences in DAG substrate specificities between *C. sativa* DGAT1 and DGAT2 are most likely the cause for the different background activities seen in the assays with the two enzymes, as previously described for the DGATs from Crambe.

### The Acyl Donor (PC) Specificity of the PDAT Enzyme Varies Depending on the DAG Acyl Acceptor Species

Overexpression of *C. sativa* PDAT in *C. sativa* seeds led to a substantial increase in the relative proportions of 18:2 in seed TAG on the expense of 18:3 and 20:1 (Marmon et al., 2017). In order to see if these changes could be correlated to the substrate specificities of *C. sativa* PDAT, we assayed PDAT activity and specificity in microsomal preparations from developing seeds of the PDAT overexpressor.

To our knowledge, there are only, three published studies regarding the substrate specificities of PDAT in plants (Dahlqvist et al., 2000; Ståhl et al., 2004; Banaś et al., 2013) and one in yeast (Ghosal et al., 2007), all performed in microsomal fractions. In all these reports, assays were done with PC carrying different radioactive acyl groups at position *sn*-2 in combination with added di-18:1-DAG. The plant PDAT enzymes (from *Arabidopsis*, sunflower, and safflower) showed much higher activity with 18:2 and 18:3 than with 18:1 at the *sn*-2 position of PC as acyl donor, whereas the yeast enzyme had about the same activity with all three acyl groups. *In vivo* feeding of 18:3 FAs to yeast expressing two linseed PDATs indicated that these enzymes had high specificity for linolenoyl containing substrates (Pan et al., 2013). Furthermore, high PDAT activities with ricinoleoyl-PC and vernoleoyl-PC were seen in microsomal preparations from developing seeds of castor bean and *Crepis palaestinae*, respectively (Dahlqvist et al., 2000). Castor bean and *C. palaestinae* accumulate high amounts of ricinoleoyl and vernoleoyl acyl groups, respectively, in their TAGs. It was later shown that expression of a castor bean PDAT in *Arabidopsis* expressing the castor bean Δ^12^hydroxylase significantly increased the content of ricinoleoyl groups in seed TAG (van Erp et al., 2011).

Due to a very good *in vitro* activity of PDAT in microsomal preparations from *C. sativa* seeds overexpressing its own PDAT and the substantial increase in activity seen by exogenous added DAG, we could study the combinatory effects of different acyl-PC and DAG species as substrates. This is the first report of both acyl donor and acyl acceptor specificities of a PDAT enzyme. The preference of 18:2-PC and 18:3-PC over 18:1-PC that was found in previous assays of plant PDATs (Ståhl et al., 2004; Banaś et al., 2013) was also seen in *C. sativa* PDAT with all DAG species tested (di-18:1-DAG, di-18:2-DAG, and di-18:3 DAG). The combination of 18:1-PC and di-18:1-DAG did not differ significantly from background activity without the addition of DAG, indicating a negligible PDAT activity with this substrate combination. Unexpectedly, the acyl acceptor molecular species influenced the specificity of the acyl donor species differently. With di-18:2-DAG, 18:3-PC was preferred over 18:2-PC, whereas the opposite preference was seen with di-18:3-DAG (**Figure 4**).

Overexpression of PDAT in *C. sativa* seeds led to an increase in mainly 54:7 and 54:8 TAG species compared to wildtype (Marmon et al., 2017). Considering the substrate specificity of *C. sativa* PDAT and that di-18:2-DAG and di-18:3-DAG are major DAG species in *C. sativa* developing seeds (Marmon et al., 2017), we can predict that PDAT would primarily produce these TAG species (i.e., 18:2/18:2/18:3 and 18:3/18:3/18:2) in the seeds. It should be noted that downregulation of PDAT in *C. sativa* with ami-PDAT led to a slightly changed FA composition of seed TAG to that of wildtype with a small, but significant, decrease in 18:2 and increase in 18:1 and 20:1 (Marmon et al., 2017). This indicates that PDAT does play a role in regulating the FA composition also in wildtype *C. sativa* seeds. Contrary to *C. sativa*, a mutation of the *Arabidopsis* PDAT gene, as well as overexpression, did not show any altered FA profile in TAG compared to wildtype (Ståhl et al., 2004; Mhaske et al., 2005). However, no detailed analyses of changes in TAG species were performed in those plants. Downregulation of PDAT in a DGAT1 *Arabidopsis* mutant background led to a drastically decreased TAG accumulation and abnormal seed development (Zhang et al., 2009). Whether this essential role of PDAT in TAG formation in the DGAT1 mutant background is possibly due to an increased PDAT activity as compared to wildtype *Arabidopsis* has not yet been investigated.

### Properties of *C. sativa* PDCT

PDCT catalyzes the interconversion between PC and DAG and has been shown to regulate the flux of polyunsaturated FAs from PC to DAG in *Arabidopsis* and rape seeds (Lu et al., 2009; Bai et al., 2020). Marmon et al. (2017) showed that microsomal fractions of developing *C. sativa* seeds had high PDCT activity. We measured the *C. sativa* PDCT activity in microsomal preparations prepared from yeast H1246 expressing the enzyme by following the flux of radioactivity from DAG, produced from [^14^C]G3P and different acyl-CoA, into PC. Only very small amounts of radioactivity entered PC when testing microsomal preparations from control cells or yeast cells expressing the *C. sativa* PDCT-like gene, indicating that this gene did not encode an active PDCT enzyme.

Substantial radioactivity however was found in PC in assays with microsomes from yeast expressing the *C. sativa* PDCT gene. Here, time-course incubations with [^14^C]G3P and 18:1-, 18:2-, or 18:3-CoA added showed a flux of radioactive acyl groups from DAG into PC, resulting in a larger amount of radiolabeled PC than DAG after 60 min (**Figure 6**). Due to different acylation rates of the different acyl-CoAs to the [^14^C]G3P and different conversion rates of PA into DAG, we were able to study the effect of different flux rates of radioactivity into DAG and further into PC. Using 18:2-CoA, the accumulation rate of radioactivity in DAG + PC was much lower than with 18:1 and 18:3-CoA after 60 min. The radiolabeled DAG with 18:2-CoA remained more or less constant between 30 and 60 min, whereas the radiolabeled PC increased during the whole time course, resulting in 2.7-fold more radioactivity in PC than in DAG after 60 min. This indicates that PDCT rates match well the levels of DAG produced in these incubations. In incubations with 18:1-CoA and 18:2-CoA, we observed much higher conversion rates of PA into DAG between 30 and 60 min and a substantial increase of radiolabel in both DAG and PC, indicating that PDCT rates were lower than the formation of radioactive DAG.

There was a lag period of 30 min before the radiolabeling of PC significantly exceeded the radiolabeling of DAG with all three different acyl-CoA investigated. We measured the levels of PC and endogenous DAG in these microsomes and found that PC amounted to 33 nmol and DAG to 6 nmol per assay (**Supplementary Table 5**). There is thus only a moderate increase in the total DAG pool formed from [^14^C]DAG after 60 min of incubation (at the most 30%). This argues against the idea that the total pool of endogenous DAG equilibrates with the newly formed [^14^C]DAG before the radiolabel enters PC. It rather suggests that DAG derived from PA replenishes a separate pool of DAG before efficient exchange of phosphocholine groups between PC and DAG can occur. Such a scenario is in line with *in vivo* radiolabeling studies in developing soybean seeds, where Bates et al. (2009) conclude that there is a sub-pool of DAG that enters PC during TAG biosynthesis. However, it should be noted that the endogenous DAG in H1246 microsomal membranes might be localized differently from DAG in the developing seeds.

Bates and Browse (2011) noted from *in vivo* radio-labelling studies that developing *Arabidopsis* seeds expressing the castor bean Δ^12^hydroxylase (hydroxylating oleic acid to ricinoleic acid) had a 70% reduction in the flux of DAG into PC compared to control plants. They attributed this to the presence of ricinoleoyl groups in DAG inhibiting the DAG to PC transacylation reaction. We therefore incubated yeast H1246 microsomes expressing the *C. sativa* PDCT with [^14^C]G3P and ricinoleoyl-CoA for 60 min and analyzed the radioactivity in the formed lipids. We found that significant amounts of radioactive di-ricinoleoyl-DAG were formed but no radioactive PC, indicating that PDCT could not use ricinoleoyl-DAG. In assays with different ratios of ricinoleoyl-CoA and 18:3-CoA, substantial radioactivity accumulated both in DAG with and without ricinoeloyl groups and with a much-decreased ratio of radioactivity (up 90%) between PC and DAG with no ricinoleoyl groups. Thus, the results show not only that ricinoleoyl-DAG is not used by *C. sativa* PDCT, but that these DAG species are also inhibitors for the conversion of DAG without ricinoleoyl groups by PDCT. The results are thus in-line with the inhibition of the flow of acyl groups from DAG into PC found in *Arabidopsis* expressing the castor bean hydroxylase (Bates and Browse, 2011). In this context it should be noted that Hu et al. (2012) assayed PDCT activity in microsomal preparations from yeast expressing the castor bean and *Arabidopsis* PDCTs by measuring the radioactive PC formed after adding radioactive DAG and either soybean PC or di-ricinoleoyl-PC. They found that radioactivity was transferred from DAG to PC in incubations with both PC species by both the *Arabidopsis* PDCT and castor bean PDCT, albeit at a lower extent with added rincinoleoyl-PC. Such assays would most likely also generate radioactive PC without addition of exogenous PC (Stymne, unpublished observations). No such assays were presented and without knowing the exact amounts of added PC involved in the phosphocholine exchange with the added DAG it is thus difficult to draw any conclusion regarding a PDCT substrate specificity toward PC.

It is interesting in this context to note that in the assays where ricinoleoyl-CoA and 18:3-CoA were mixed (**Figure 7B**), most of the ricinoleoyl groups accumulated as di-ricinoleoyl-DAG and not as mixed acyl-DAG species, even in incubations with ratios of 18:3-CoA to ricinoleoyl-CoA of 4:1. This indicates that the yeast LPAT enzymes acylating ricinoleoyl-LPA (SLC1 and ALE1) prefer ricinoleoyl-CoA over 18:3-CoA. In this respect they differ from the *Arabidopsis* LPAT2 which is unable to produce di-ricinoleoyl species and is more similar to the castor bean LPAT2 (Shockey et al., 2019).

In conclusion, our investigations of *C. sativa* DGATs and PDAT demonstrate the importance of studying the specificities of the enzymes with different combinations of naturally occurring acyl-donors and acceptors. Using artificial acyl acceptors, such as di-6:0-DAG, DGAT assays do not necessarily reflect the in planta DGAT specificity with natural acyl acceptors. Whether this is the case also for PDAT assays (Falarz et al., 2019) remains to be established. The substrate specificities of *C. sativa* DGATs and PDAT presented here corroborate well the changes in TAG species found in seeds of *C. sativa* with downregulated DGAT1 and upregulated PDAT expression, respectively (Marmon et al., 2017). The kinetics of the conversion of *de novo* synthesized DAG in yeast microsomes through *C. sativa* PDCT suggest that these DAG molecules do not equilibrate with the whole endogenous DAG pool before entering PC *via* the PDCT reaction. Furthermore, it was shown that ricinoleoyl-DAG species are not used by PDCT, and their presence also inhibits PDCT catalyzed transfer of ricinoleoyl-free DAG into PC.

## Data Availability Statement

The datasets presented in this study can be found in online repositories. The names of the repository/repositories and accession number(s) can be found below: https://www.ncbi.nlm.nih.gov/genbank/, KY263957, https://www.ncbi.nlm.nih.gov/genbank/, KY263958, https://www.ncbi.nlm.nih.gov/genbank/, MT130512, https://www.ncbi.nlm.nih.gov/genbank/, MT130513, https://www.ncbi.nlm.nih.gov/genbank/, MT130514.

## Author Contributions

SM, SJ, IL, IF, and SS conceived the original research plan. IL, SJ, A-LG, SM, and SS performed the experiments. SS wrote the article with contributions from all the authors.

## Funding

This work was supported by the Swedish Research Council (grant no. 637-2013-430 to SM), the Swedish Foundation for Strategic Research as a part of the project Oil Crops for the Future (RBP14-0037), the strategic research program Crops for the Future (C4F), and the German Federal Ministry of Education and Research (grant no. 031A589A to IF).

## Conflict of Interest

The authors declare that the research was conducted in the absence of any commercial or financial relationships that could be construed as a potential conflict of interest.
