# Supplementary material for: Acyltransferases Regulate Oil Quality in Camelina sativa Through Both Acyl Donor and Acyl Acceptor Specificities
Source: Front Plant Sci. 2020 Aug 14;11:1144. doi: 10.3389/fpls.2020.01144 (PMC7456936; doi:10.3389/fpls.2020.01144)
Supplement: Supplementary file 1 [file DataSheet_1.pdf]

Supplementary Material

Supplementary Table 1. DGAT activtiy with no DAG added (DMSO background). Standard deviation (SD) in paranthesis (n=3 replicates).

	nmol TAG/min/mg protein		
	18:1-CoA	18:2-CoA	18:3-CoA
DGAT1	0.58 (0.05)	1.16 (0.39)	0.88 (0.24)
DGAT2	0	0	0

Supplementary Table 2. Statistical analysis of the differences in activity of *C. sativa* DGAT1 between different combinations of Acyl-CoA and DAG substrates. Stars indicate significance, * p ≤ 0,05, ** p ≤ 0,01 and *** p ≤ 0,001, as determined by one-way ANOVA followed by Tukeys test (n=3 replicates).

Species compared (average nmol TAG/mgprotein/min formed)	p-value	Significance
18:1-CoA_16:0/18:2-DAG (7,0) 18:1-CoA_di-18:1-DAG (4,6)	0,135	
18:1-CoA_16:0/18:2-DAG (7,0) 18:1-CoA_di-18:2-DAG (0,3)	8,97E-08	***
18:1-CoA_16:0/18:2-DAG (7,0) 18:1-CoA_di-18:3-DAG (0,6)	1,48E-07	***
18:1-CoA_16:0/18:2-DAG (7,0) 18:2-CoA_di-18:1-DAG (11,5)	7,21E-05	***
18:1-CoA_16:0/18:2-DAG (7,0) 18:2-CoA_di-18:2-DAG (0,7)	1,84E-07	***
18:1-CoA_16:0/18:2-DAG (7,0) 18:2-CoA_di-18:3-DAG (1,2)	7,21E-07	***
18:1-CoA_16:0/18:2-DAG (7,0) 18:3-CoA_di-18:1-DAG (10,4)	0,00371	**
18:1-CoA_16:0/18:2-DAG (7,0) 18:3-CoA_di-18:2-DAG (0,6)	1,32E-07	***
18:1-CoA_16:0/18:2-DAG (7,0) 18:3-CoA_di-18:3-DAG (1,0)	3,24E-07	***
18:1-CoA_18:2/16:0-DAG (13) 18:1-CoA_16:0/18:2-DAG (7,0)	1,83E-07	***
18:1-CoA_18:2/16:0-DAG (13) 18:1-CoA_di-18:1-DAG (4,6)	1,44E-06	***
18:1-CoA_18:2/16:0-DAG (13) 18:1-CoA_di-18:2-DAG (0,3)	0	***
18:1-CoA_18:2/16:0-DAG (13) 18:1-CoA_di-18:3-DAG (0,6)	0	***
18:1-CoA_18:2/16:0-DAG (13) 18:2-CoA_16:0/18:2-DAG (10,1)	0,0111	*
18:1-CoA_18:2/16:0-DAG (13) 18:2-CoA_di-18:1-DAG (11,5)	0,542	
18:1-CoA_18:2/16:0-DAG (13) 18:2-CoA_di-18:2-DAG (0,7)	0	***
18:1-CoA_18:2/16:0-DAG (13) 18:2-CoA_di-18:3-DAG (1,2)	0	***
18:1-CoA_18:2/16:0-DAG (13) 18:3-CoA_16:0/18:2-DAG (7,9)	3,80E-06	***
18:1-CoA_18:2/16:0-DAG (13) 18:3-CoA_di-18:1-DAG (10,4)	0,0344	*
18:1-CoA_18:2/16:0-DAG (13) 18:3-CoA_di-18:2-DAG (0,6)	0	***
18:1-CoA_18:2/16:0-DAG (13) 18:3-CoA_di-18:3-DAG (1,0)	0	***
18:1-CoA_di-18:2-DAG (0,3) 18:1-CoA_di-18:1-DAG (4,6)	1,33E-04	***
18:1-CoA_di-18:2-DAG (0,3) 18:2-CoA_di-18:1-DAG (11,5)	5,85E-10	***
18:1-CoA_di-18:2-DAG (0,3) 18:3-CoA_di-18:1-DAG (10,4)	5,51E-08	***
18:1-CoA_di-18:3-DAG (0,6) 18:1-CoA_di-18:1-DAG (4,6)	4,86E-04	***
18:1-CoA_di-18:3-DAG (0,6) 18:1-CoA_di-18:2-DAG (0,3)	1	
18:1-CoA_di-18:3-DAG (0,6) 18:2-CoA_di-18:1-DAG (11,5)	3,22E-09	***
18:1-CoA_di-18:3-DAG (0,6) 18:2-CoA_di-18:2-DAG (0,7)	1	
18:1-CoA_di-18:3-DAG (0,6) 18:3-CoA_di-18:1-DAG (10,4)	4,57E-08	***
18:1-CoA_di-18:3-DAG (0,6) 18:3-CoA_di-18:2-DAG (0,6)	1	
18:2-CoA_16:0/18:2-DAG (10,1) 18:1-CoA_16:0/18:2-DAG (7,0)	0,0121	*
18:2-CoA_16:0/18:2-DAG (10,1) 18:1-CoA_di-18:1-DAG (4,6)	2,16E-06	***
18:2-CoA_16:0/18:2-DAG (10,1) 18:1-CoA_di-18:2-DAG (0,3)	4,56E-08	***
18:2-CoA_16:0/18:2-DAG (10,1) 18:1-CoA_di-18:3-DAG (0,6)	4,76E-08	***
18:2-CoA_16:0/18:2-DAG (10,1) 18:2-CoA_di-18:1-DAG (11,5)	0,828	
18:2-CoA_16:0/18:2-DAG (10,1) 18:2-CoA_di-18:2-DAG (0,7)	4,82E-08	***
18:2-CoA_16:0/18:2-DAG (10,1) 18:2-CoA_di-18:3-DAG (1,2)	1,45E-07	***

18:2-CoA_16:0/18:2-DAG (10,1) 18:3-CoA_di-18:1-DAG (10,4)	1	
18:2-CoA_16:0/18:2-DAG (10,1) 18:3-CoA_di-18:2-DAG (0,6)	4,73E-08	***
18:2-CoA_16:0/18:2-DAG (10,1) 18:3-CoA_di-18:3-DAG (1,0)	4,96E-08	***
18:2-CoA_18:2/16:0-DAG (15) 18:1-CoA_16:0/18:2-DAG (7,0)	1,29E-07	***
18:2-CoA_18:2/16:0-DAG (15) 18:1-CoA_18:2/16:0-DAG (13)	0,775	
18:2-CoA_18:2/16:0-DAG (15) 18:1-CoA_di-18:1-DAG (4,6)	5,52E-08	***
18:2-CoA_18:2/16:0-DAG (15) 18:1-CoA_di-18:2-DAG (0,3)	0	***
18:2-CoA_18:2/16:0-DAG (15) 18:1-CoA_di-18:3-DAG (0,6)	0	***
18:2-CoA_18:2/16:0-DAG (15) 18:2-CoA_16:0/18:2-DAG (10,1)	5,03E-05	***
18:2-CoA_18:2/16:0-DAG (15) 18:2-CoA_di-18:1-DAG (11,5)	0,00864	**
18:2-CoA_18:2/16:0-DAG (15) 18:2-CoA_di-18:2-DAG (0,7)	0	***
18:2-CoA_18:2/16:0-DAG (15) 18:2-CoA_di-18:3-DAG (1,2)	0	***
18:2-CoA_18:2/16:0-DAG (15) 18:3-CoA_16:0/18:2-DAG (7,9)	8,31E-08	***
18:2-CoA_18:2/16:0-DAG (15) 18:3-CoA_di-18:1-DAG (10,4)	1,74E-04	***
18:2-CoA_18:2/16:0-DAG (15) 18:3-CoA_di-18:2-DAG (0,6)	0	***
18:2-CoA_18:2/16:0-DAG (15) 18:3-CoA_di-18:3-DAG (1,0)	0	***
18:2-CoA_di-18:1-DAG (11,5) 18:1-CoA_di-18:1-DAG (4,6)	7,75E-08	***
18:2-CoA_di-18:2-DAG (0,7) 18:1-CoA_di-18:1-DAG (4,6)	7,22E-04	***
18:2-CoA_di-18:2-DAG (0,7) 18:1-CoA_di-18:2-DAG (0,3)	1	
18:2-CoA_di-18:2-DAG (0,7) 18:2-CoA_di-18:1-DAG (11,5)	4,03E-09	***
18:2-CoA_di-18:2-DAG (0,7) 18:3-CoA_di-18:1-DAG (10,4)	4,63E-08	***
18:2-CoA_di-18:3-DAG (1,2) 18:1-CoA_di-18:1-DAG (4,6)	0,00407	**
18:2-CoA_di-18:3-DAG (1,2) 18:1-CoA_di-18:2-DAG (0,3)	0,992	
18:2-CoA_di-18:3-DAG (1,2) 18:1-CoA_di-18:3-DAG (0,6)	1,00	
18:2-CoA_di-18:3-DAG (1,2) 18:2-CoA_di-18:1-DAG (11,5)	7,64E-09	***
18:2-CoA_di-18:3-DAG (1,2) 18:2-CoA_di-18:2-DAG (0,7)	1	
18:2-CoA_di-18:3-DAG (1,2) 18:3-CoA_di-18:1-DAG (10,4)	4,90E-08	***
18:2-CoA_di-18:3-DAG (1,2) 18:3-CoA_di-18:2-DAG (0,6)	1,00	
18:3-CoA_16:0/18:2-DAG (7,9) 18:1-CoA_16:0/18:2-DAG (7,0)	0,991	
18:3-CoA_16:0/18:2-DAG (7,9) 18:1-CoA_di-18:1-DAG (4,6)	0,00639	**
18:3-CoA_16:0/18:2-DAG (7,9) 18:1-CoA_di-18:2-DAG (0,3)	1,27E-07	***
18:3-CoA_16:0/18:2-DAG (7,9) 18:1-CoA_di-18:3-DAG (0,6)	0	***
18:3-CoA_16:0/18:2-DAG (7,9) 18:2-CoA_16:0/18:2-DAG (10,1)	0,219	
18:3-CoA_16:0/18:2-DAG (7,9) 18:2-CoA_di-18:1-DAG (11,5)	0,00237	**
18:3-CoA_16:0/18:2-DAG (7,9) 18:2-CoA_di-18:2-DAG (0,7)	6,53E-08	***
18:3-CoA_16:0/18:2-DAG (7,9) 18:2-CoA_di-18:3-DAG (1,2)	8,85E-08	***
18:3-CoA_16:0/18:2-DAG (7,9) 18:3-CoA_di-18:1-DAG (10,4)	0,0875	
18:3-CoA_16:0/18:2-DAG (7,9) 18:3-CoA_di-18:2-DAG (0,6)	1,24E-07	***
18:3-CoA_16:0/18:2-DAG (7,9) 18:3-CoA_di-18:3-DAG (1,0)	7,35E-08	***
18:3-CoA_18:2/16:0-DAG (17) 18:1-CoA_16:0/18:2-DAG (7,0)	4,67E-08	***
18:3-CoA_18:2/16:0-DAG (17) 18:1-CoA_18:2/16:0-DAG (13)	0,00463	**
18:3-CoA_18:2/16:0-DAG (17) 18:1-CoA_di-18:1-DAG (4,6)	0	***
18:3-CoA_18:2/16:0-DAG (17) 18:1-CoA_di-18:2-DAG (0,3)	0	***
18:3-CoA_18:2/16:0-DAG (17) 18:1-CoA_di-18:3-DAG (0,6)	0	***
18:3-CoA_18:2/16:0-DAG (17) 18:2-CoA_16:0/18:2-DAG (10,1)	1,12E-07	***

18:3-CoA_18:2/16:0-DAG (17) 18:2-CoA_18:2/16:0-DAG (15)	0,393	
18:3-CoA_18:2/16:0-DAG (17) 18:2-CoA_di-18:1-DAG (11,5)	7,42E-06	***
18:3-CoA_18:2/16:0-DAG (17) 18:2-CoA_di-18:2-DAG (0,7)	0	***
18:3-CoA_18:2/16:0-DAG (17) 18:2-CoA_di-18:3-DAG (1,2)	0	***
18:3-CoA_18:2/16:0-DAG (17) 18:3-CoA_16:0/18:2-DAG (7,9)	1,42E-07	***
18:3-CoA_18:2/16:0-DAG (17) 18:3-CoA_di-18:1-DAG (10,4)	2,11E-07	***
18:3-CoA_18:2/16:0-DAG (17) 18:3-CoA_di-18:2-DAG (0,6)	0	***
18:3-CoA_18:2/16:0-DAG (17) 18:3-CoA_di-18:3-DAG (1,0)	0	***
18:3-CoA_di-18:1-DAG (10,4) 18:1-CoA_di-18:1-DAG (4,6)	6,61E-07	***
18:3-CoA_di-18:1-DAG (10,4) 18:2-CoA_di-18:1-DAG (11,5)	0,973	
18:3-CoA_di-18:2-DAG (0,6) 18:1-CoA_di-18:1-DAG (4,6)	3,86E-04	***
18:3-CoA_di-18:2-DAG (0,6) 18:1-CoA_di-18:2-DAG (0,3)	1	
18:3-CoA_di-18:2-DAG (0,6) 18:2-CoA_di-18:1-DAG (11,5)	2,75E-09	***
18:3-CoA_di-18:2-DAG (0,6) 18:2-CoA_di-18:2-DAG (0,7)	1	
18:3-CoA_di-18:2-DAG (0,6) 18:3-CoA_di-18:1-DAG (10,4)	4,53E-08	***
18:3-CoA_di-18:3-DAG (1,0) 18:1-CoA_di-18:1-DAG (4,6)	0,00177	**
18:3-CoA_di-18:3-DAG (1,0) 18:1-CoA_di-18:2-DAG (0,3)	1,00	
18:3-CoA_di-18:3-DAG (1,0) 18:1-CoA_di-18:3-DAG (0,6)	1	
18:3-CoA_di-18:3-DAG (1,0) 18:2-CoA_di-18:1-DAG (11,5)	5,89E-09	***
18:3-CoA_di-18:3-DAG (1,0) 18:2-CoA_di-18:2-DAG (0,7)	1	
18:3-CoA_di-18:3-DAG (1,0) 18:2-CoA_di-18:3-DAG (1,2)	1	
18:3-CoA_di-18:3-DAG (1,0) 18:3-CoA_di-18:1-DAG (10,4)	4,77E-08	***
18:3-CoA_di-18:3-DAG (1,0) 18:3-CoA_di-18:2-DAG (0,6)	1	

Supplementary Table 3. Statistical analysis of the differences in activity of *C. sativa* DGAT2 between different combinations of Acyl-CoA and DAG substrates. Stars indicate significance, * $p \leq 0,05$, ** $p \leq 0,01$ and *** $p \leq 0,001$, as determined by one-way ANOVA followed by Tukeys test (n=3 replicates).

Species compared (average nmol TAG/mgprotein/min formed)	p-value	Significance
18:1-CoA_16:0/18:2-DAG (0,01) 18:1-CoA_di-18:1-DAG (0,02)	1	
18:1-CoA_18:2/16:0-DAG (0,01) 18:1-CoA_di-18:1-DAG (0,02)	1	
18:1-CoA_di-18:2-DAG (0,14) 18:1-CoA_di-18:1-DAG (0,02)	0,697	
18:1-CoA_di-18:3-DAG (0,59) 18:1-CoA_di-18:1-DAG (0,02)	1,24E-07	***
18:1-CoA_16:0/18:2-DAG (0,01) 18:1-CoA_di-18:2-DAG (0,14)	0,582	
18:1-CoA_16:0/18:2-DAG (0,01) 18:1-CoA_di-18:3-DAG (0,59)	1,26E-07	***
18:1-CoA_18:2/16:0-DAG (0,01) 18:1-CoA_16:0/18:2-DAG (0,01)	1	
18:1-CoA_18:2/16:0-DAG (0,01) 18:1-CoA_di-18:2-DAG (0,14)	0,597	
18:1-CoA_18:2/16:0-DAG (0,01) 18:1-CoA_di-18:3-DAG (0,59)	1,25E-07	***
18:1-CoA_di-18:3-DAG (0,59) 18:1-CoA_di-18:2-DAG (0,14)	7,18E-07	***
18:1-CoA_16:0/18:2-DAG (0,01) 18:2-CoA_di-18:1-DAG (0,12)	0,797	
18:1-CoA_18:2/16:0-DAG (0,01) 18:2-CoA_di-18:1-DAG (0,12)	0,809	
18:1-CoA_di-18:2-DAG (0,14) 18:2-CoA_di-18:1-DAG (0,12)	1	
18:1-CoA_di-18:3-DAG (0,59) 18:2-CoA_di-18:1-DAG (0,12)	3,10E-07	***
18:1-CoA_16:0/18:2-DAG (0,01) 18:2-CoA_di-18:2-DAG (1,2)	0	***
18:1-CoA_16:0/18:2-DAG (0,01) 18:2-CoA_di-18:3-DAG (1,4)	0	***
18:1-CoA_18:2/16:0-DAG (0,01) 18:2-CoA_16:0/18:2-DAG (0,07)	0,999	
18:1-CoA_18:2/16:0-DAG (0,01) 18:2-CoA_di-18:2-DAG (1,2)	0	***
18:1-CoA_18:2/16:0-DAG (0,01) 18:2-CoA_di-18:3-DAG (1,4)	0	***
18:1-CoA_di-18:3-DAG (0,59) 18:2-CoA_di-18:2-DAG (1,2)	3,21E-07	***
18:1-CoA_16:0/18:2-DAG (0,01) 18:3-CoA_di-18:1-DAG (0,13)	0,727	
18:1-CoA_16:0/18:2-DAG (0,01) 18:3-CoA_di-18:2-DAG (2,0)	4,65E-07	***
18:1-CoA_16:0/18:2-DAG (0,01) 18:3-CoA_di-18:3-DAG (1,6)	0	***
18:1-CoA_18:2/16:0-DAG (0,01) 18:3-CoA_16:0/18:2-DAG (0,10)	0,947	
18:1-CoA_18:2/16:0-DAG (0,01) 18:3-CoA_di-18:1-DAG (0,13)	0,740	
18:1-CoA_18:2/16:0-DAG (0,01) 18:3-CoA_di-18:2-DAG (2,0)	4,64E-07	***
18:1-CoA_18:2/16:0-DAG (0,01) 18:3-CoA_di-18:3-DAG (1,6)	0	***
18:1-CoA_di-18:2-DAG (0,14) 18:3-CoA_di-18:1-DAG (0,13)	1	
18:1-CoA_di-18:3-DAG (0,59) 18:3-CoA_di-18:1-DAG (0,13)	4,12E-07	***
18:1-CoA_di-18:3-DAG (0,59) 18:3-CoA_di-18:2-DAG (2,0)	0	***
18:2-CoA_di-18:1-DAG (0,12) 18:1-CoA_di-18:1-DAG (0,02)	0,882	
18:2-CoA_16:0/18:2-DAG (0,07) 18:1-CoA_di-18:1-DAG (0,02)	1,00	
18:2-CoA_18:2/16:0-DAG (0,02) 18:1-CoA_di-18:1-DAG (0,02)	0,999	
18:2-CoA_di-18:2-DAG (1,2) 18:1-CoA_di-18:1-DAG (0,02)	0	***
18:2-CoA_di-18:3-DAG (1,4) 18:1-CoA_di-18:1-DAG (0,02)	0	***
18:2-CoA_16:0/18:2-DAG (0,07) 18:1-CoA_16:0/18:2-DAG (0,01)	0,999	
18:2-CoA_16:0/18:2-DAG (0,07) 18:1-CoA_di-18:2-DAG (0,14)	0,990	
18:2-CoA_16:0/18:2-DAG (0,07) 18:1-CoA_di-18:3-DAG (0,59)	8,81E-08	***

18:2-CoA_18:2/16:0-DAG (0,02) 18:1-CoA_16:0/18:2-DAG (0,01)	0,994	
18:2-CoA_18:2/16:0-DAG (0,02) 18:1-CoA_18:2/16:0-DAG (0,01)	0,995	
18:2-CoA_18:2/16:0-DAG (0,02) 18:1-CoA_di-18:2-DAG (0,14)	0,998	
18:2-CoA_18:2/16:0-DAG (0,02) 18:1-CoA_di-18:3-DAG (0,59)	1,03E-07	***
18:2-CoA_di-18:2-DAG (1,2) 18:1-CoA_di-18:2-DAG (0,14)	0	***
18:2-CoA_di-18:3-DAG (1,4) 18:1-CoA_di-18:2-DAG (0,14)	0	
18:2-CoA_di-18:3-DAG (1,4) 18:1-CoA_di-18:3-DAG (0,59)	4,01E-09	***
18:2-CoA_16:0/18:2-DAG (0,07) 18:2-CoA_di-18:1-DAG (0,12)	1,00	
18:2-CoA_18:2/16:0-DAG (0,02) 18:2-CoA_di-18:1-DAG (0,12)	1,00	
18:2-CoA_di-18:2-DAG (1,2) 18:2-CoA_di-18:1-DAG (0,12)	0	***
18:2-CoA_di-18:3-DAG (1,4) 18:2-CoA_di-18:1-DAG (0,12)	0	***
18:2-CoA_16:0/18:2-DAG (0,07) 18:2-CoA_di-18:2-DAG (1,2)	0	***
18:2-CoA_16:0/18:2-DAG (0,07) 18:2-CoA_di-18:3-DAG (1,4)	0	***
18:2-CoA_18:2/16:0-DAG (0,02) 18:2-CoA_16:0/18:2-DAG (0,07)	1	
18:2-CoA_18:2/16:0-DAG (0,02) 18:2-CoA_di-18:2-DAG (1,2)	0	***
18:2-CoA_18:2/16:0-DAG (0,02) 18:2-CoA_di-18:3-DAG (1,4)	0	***
18:2-CoA_di-18:3-DAG (1,4) 18:2-CoA_di-18:2-DAG (1,2)	0,0433	***
18:2-CoA_16:0/18:2-DAG (0,07) 18:3-CoA_di-18:1-DAG (0,13)	0,999	
18:2-CoA_16:0/18:2-DAG (0,07) 18:3-CoA_di-18:2-DAG (2,0)	0	***
18:2-CoA_16:0/18:2-DAG (0,07) 18:3-CoA_di-18:3-DAG (1,6)	0	***
18:2-CoA_18:2/16:0-DAG (0,02) 18:3-CoA_16:0/18:2-DAG (0,10)	1	
18:2-CoA_18:2/16:0-DAG (0,02) 18:3-CoA_di-18:1-DAG (0,13)	1,00	
18:2-CoA_18:2/16:0-DAG (0,02) 18:3-CoA_di-18:2-DAG (2,0)	0	***
18:2-CoA_18:2/16:0-DAG (0,02) 18:3-CoA_di-18:3-DAG (1,6)	0	***
18:2-CoA_di-18:2-DAG (1,2) 18:3-CoA_di-18:1-DAG (0,13)	0	***
18:2-CoA_di-18:3-DAG (1,4) 18:3-CoA_di-18:1-DAG (0,13)	0	***
18:2-CoA_di-18:3-DAG (1,4) 18:3-CoA_di-18:2-DAG (2,0)	8,27E-08	***
18:3-CoA_16:0/18:2-DAG (0,10) 18:1-CoA_di-18:1-DAG (0,02)	0,976	
18:3-CoA_di-18:1-DAG (0,13) 18:1-CoA_di-18:1-DAG (0,02)	0,826	
18:3-CoA_18:2/16:0-DAG (0,07) 18:1-CoA_di-18:1-DAG (0,02)	1,00	
18:3-CoA_di-18:2-DAG (2,0) 18:1-CoA_di-18:1-DAG (0,02)	4,59E-07	***
18:3-CoA_di-18:3-DAG (1,6) 18:1-CoA_di-18:1-DAG (0,02)	0	***
18:3-CoA_16:0/18:2-DAG (0,10) 18:1-CoA_16:0/18:2-DAG (0,01)	0,941	
18:3-CoA_16:0/18:2-DAG (0,10) 18:1-CoA_di-18:2-DAG (0,14)	1,00	
18:3-CoA_16:0/18:2-DAG (0,10) 18:1-CoA_di-18:3-DAG (0,59)	1,68E-07	***
18:3-CoA_18:2/16:0-DAG (0,07) 18:1-CoA_16:0/18:2-DAG (0,01)	0,999	
18:3-CoA_18:2/16:0-DAG (0,07) 18:1-CoA_18:2/16:0-DAG (0,01)	0,999	
18:3-CoA_18:2/16:0-DAG (0,07) 18:1-CoA_di-18:2-DAG (0,14)	0,990	
18:3-CoA_18:2/16:0-DAG (0,07) 18:1-CoA_di-18:3-DAG (0,59)	8,77E-08	***
18:3-CoA_di-18:2-DAG (2,0) 18:1-CoA_di-18:2-DAG (0,14)	0	***
18:3-CoA_di-18:3-DAG (1,6) 18:1-CoA_di-18:2-DAG (0,14)	0	***
18:3-CoA_di-18:3-DAG (1,6) 18:1-CoA_di-18:3-DAG (0,59)	0	***
18:3-CoA_16:0/18:2-DAG (0,10) 18:2-CoA_di-18:1-DAG (0,12)	1	
18:3-CoA_di-18:1-DAG (0,13) 18:2-CoA_di-18:1-DAG (0,12)	1	

18:3-CoA_18:2/16:0-DAG (0,07) 18:2-CoA_di-18:1-DAG (0,12)	1,00	
18:3-CoA_di-18:2-DAG (2,0) 18:2-CoA_di-18:1-DAG (0,12)	0	***
18:3-CoA_di-18:3-DAG (1,6) 18:2-CoA_di-18:1-DAG (0,12)	0	***
18:3-CoA_16:0/18:2-DAG (0,10) 18:2-CoA_16:0/18:2-DAG (0,07)	1	
18:3-CoA_16:0/18:2-DAG (0,10) 18:2-CoA_di-18:2-DAG (1,2)	0	***
18:3-CoA_16:0/18:2-DAG (0,10) 18:2-CoA_di-18:3-DAG (1,4)	0	***
18:3-CoA_18:2/16:0-DAG (0,07) 18:2-CoA_16:0/18:2-DAG (0,07)	1	
18:3-CoA_18:2/16:0-DAG (0,07) 18:2-CoA_18:2/16:0-DAG (0,02)	1	
18:3-CoA_18:2/16:0-DAG (0,07) 18:2-CoA_di-18:2-DAG (1,2)	0	***
18:3-CoA_18:2/16:0-DAG (0,07) 18:2-CoA_di-18:3-DAG (1,4)	0	***
18:3-CoA_di-18:2-DAG (2,0) 18:2-CoA_di-18:2-DAG (1,2)	4,73E-08	***
18:3-CoA_di-18:3-DAG (1,6) 18:2-CoA_di-18:2-DAG (1,2)	1,80E-05	***
18:3-CoA_di-18:3-DAG (1,6) 18:2-CoA_di-18:3-DAG (1,4)	0,230	
18:3-CoA_16:0/18:2-DAG (0,10) 18:3-CoA_di-18:1-DAG (0,13)	1	
18:3-CoA_16:0/18:2-DAG (0,10) 18:3-CoA_di-18:2-DAG (2,0)	1,38E-06	***
18:3-CoA_16:0/18:2-DAG (0,10) 18:3-CoA_di-18:3-DAG (1,6)	0	***
18:3-CoA_18:2/16:0-DAG (0,07) 18:3-CoA_16:0/18:2-DAG (0,10)	1	
18:3-CoA_18:2/16:0-DAG (0,07) 18:3-CoA_di-18:1-DAG (0,13)	0,998	
18:3-CoA_18:2/16:0-DAG (0,07) 18:3-CoA_di-18:2-DAG (2,0)	0	***
18:3-CoA_18:2/16:0-DAG (0,07) 18:3-CoA_di-18:3-DAG (1,6)	0	***
18:3-CoA_di-18:2-DAG (2,0) 18:3-CoA_di-18:1-DAG (0,13)	0	***
18:3-CoA_di-18:3-DAG (1,6) 18:3-CoA_di-18:1-DAG (0,13)	0	***
18:3-CoA_di-18:3-DAG (1,6) 18:3-CoA_di-18:2-DAG (2,0)	4,57E-05	***

Supplementary Table 4. Statistical analysis of the differences in activity of *C. sativa* PDAT between different combinations of PC and DAG substrates. Stars indicate significance, * p ≤ 0,05, ** p ≤ 0,01 and *** p ≤ 0,001, as determined by one-way ANOVA followed by Tukeys test (n=3 replicates).

Species compared (average pmol TAG formed)	p-value	Significance
18:1-PC/di-18:2-DAG (173) 18:1-PC/di-18:1-DAG (72)	0,389	
18:1-PC/di-18:3-DAG (137) 18:1-PC/di-18:1-DAG (72)	0,899	
18:1-PC/no-DAG (77) 18:1-PC/di-18:1-DAG (72)	1	
18:2-PC/di-18:1-DAG (405) 18:1-PC/di-18:1-DAG (72)	2,04E-07	***
18:2-PC/di-18:2-DAG (257) 18:1-PC/di-18:1-DAG (72)	0,000508	***
18:2-PC/di-18:2-DAG (257) 18:2-PC/di-18:1-DAG (405)	0,00941	**
18:2-PC/di-18:3-DAG (397) 18:1-PC/di-18:1-DAG (72)	3,14E-07	***
18:2-PC/di-18:3-DAG (397) 18:2-PC/di-18:1-DAG (405)	1	
18:2-PC/no-DAG (89) 18:1-PC/di-18:1-DAG (72)	1	
18:2-PC/no-DAG (89) 18:2-PC/di-18:1-DAG (405)	5,24E-07	***
18:3-PC/di-18:1-DAG (243) 18:1-PC/di-18:1-DAG (72)	0,00955	**
18:3-PC/di-18:1-DAG (243) 18:2-PC/di-18:1-DAG (405)	0,0159	*
18:3-PC/di-18:2-DAG (419) 18:1-PC/di-18:1-DAG (72)	1,28E-07	***
18:3-PC/di-18:2-DAG (419) 18:2-PC/di-18:1-DAG (405)	1	
18:3-PC/di-18:2-DAG (419) 18:3-PC/di-18:1-DAG (243)	0,00675	**
18:3-PC/di-18:3-DAG (238) 18:1-PC/di-18:1-DAG (72)	0,00237	**
18:3-PC/di-18:3-DAG (238) 18:2-PC/di-18:1-DAG (405)	0,00213	**
18:3-PC/di-18:3-DAG (238) 18:3-PC/di-18:1-DAG (243)	1	
18:3-PC/no-DAG (53) 18:1-PC/di-18:1-DAG (72)	1,00	
18:3-PC/no-DAG (53) 18:2-PC/di-18:1-DAG (405)	9,04E-08	***
18:3-PC/no-DAG (53) 18:3-PC/di-18:1-DAG (243)	0,000349	***
18:1-PC/di-18:3-DAG (137) 18:1-PC/di-18:2-DAG (173)	0,999	
18:1-PC/no-DAG (77) 18:1-PC/di-18:2-DAG (173)	0,468	
18:2-PC/di-18:1-DAG (405) 18:1-PC/di-18:2-DAG (173)	0,000144	***
18:2-PC/di-18:2-DAG (257) 18:1-PC/di-18:2-DAG (173)	0,453	
18:2-PC/di-18:3-DAG (397) 18:1-PC/di-18:2-DAG (173)	0,000253	***
18:2-PC/di-18:3-DAG (397) 18:2-PC/di-18:2-DAG (257)	0,0170	*
18:2-PC/no-DAG (89) 18:1-PC/di-18:2-DAG (173)	0,658	
18:2-PC/no-DAG (89) 18:2-PC/di-18:2-DAG (257)	0,00202	**
18:3-PC/di-18:1-DAG (243) 18:1-PC/di-18:2-DAG (173)	0,859	
18:3-PC/di-18:1-DAG (243) 18:2-PC/di-18:2-DAG (257)	1	
18:3-PC/di-18:2-DAG (419) 18:1-PC/di-18:2-DAG (173)	5,64E-05	***
18:3-PC/di-18:2-DAG (419) 18:2-PC/di-18:2-DAG (257)	0,0034	**
18:3-PC/di-18:3-DAG (238) 18:1-PC/di-18:2-DAG (173)	0,794	
18:3-PC/di-18:3-DAG (238) 18:2-PC/di-18:2-DAG (257)	1,00	

18:3-PC/di-18:3-DAG (238) 18:3-PC/di-18:2-DAG (419)	0,000739	***
18:3-PC/no-DAG (53) 18:1-PC/di-18:2-DAG (173)	0,06435	
18:3-PC/no-DAG (53) 18:2-PC/di-18:2-DAG (257)	2,59E-06	***
18:3-PC/no-DAG (53) 18:3-PC/di-18:2-DAG (419)	8,79E-08	***
18:1-PC/no-DAG (77) 18:1-PC/di-18:3-DAG (137)	0,940	
18:2-PC/di-18:1-DAG (405) 18:1-PC/di-18:3-DAG (137)	1,15E-05	***
18:2-PC/di-18:2-DAG (257) 18:1-PC/di-18:3-DAG (137)	0,0672	
18:2-PC/di-18:3-DAG (397) 18:1-PC/di-18:3-DAG (137)	2,01E-05	***
18:2-PC/no-DAG (89) 18:1-PC/di-18:3-DAG (137)	0,988	
18:2-PC/no-DAG (89) 18:2-PC/di-18:3-DAG (397)	8,15E-07	***
18:3-PC/di-18:1-DAG (243) 18:1-PC/di-18:3-DAG (137)	0,334	
18:3-PC/di-18:1-DAG (243) 18:2-PC/di-18:3-DAG (397)	0,0262	*
18:3-PC/di-18:2-DAG (419) 18:1-PC/di-18:3-DAG (137)	4,53E-06	***
18:3-PC/di-18:2-DAG (419) 18:2-PC/di-18:3-DAG (397)	1,00	
18:3-PC/di-18:3-DAG (238) 18:1-PC/di-18:3-DAG (137)	0,210	
18:3-PC/di-18:3-DAG (238) 18:2-PC/di-18:3-DAG (397)	0,00397	**
18:3-PC/no-DAG (53) 18:1-PC/di-18:3-DAG (137)	0,441	
18:3-PC/no-DAG (53) 18:2-PC/di-18:3-DAG (397)	8,60E-08	***
18:3-PC/no-DAG (53) 18:3-PC/di-18:3-DAG (238)	1,73E-05	***
18:2-PC/di-18:1-DAG (405) 18:1-PC/no-DAG (77)	2,80E-07	***
18:2-PC/di-18:2-DAG (257) 18:1-PC/no-DAG (77)	7,80E-04	***
18:2-PC/di-18:3-DAG (397) 18:1-PC/no-DAG (77)	4,29E-07	***
18:2-PC/no-DAG (89) 18:1-PC/no-DAG (77)	1	
18:3-PC/di-18:1-DAG (243) 18:1-PC/no-DAG (77)	0,0135	*
18:3-PC/di-18:1-DAG (243) 18:2-PC/no-DAG (89)	0,0284	*
18:3-PC/di-18:2-DAG (419) 18:1-PC/no-DAG (77)	1,52E-07	***
18:3-PC/di-18:2-DAG (419) 18:2-PC/no-DAG (89)	2,66E-07	***
18:3-PC/di-18:3-DAG (238) 18:1-PC/no-DAG (77)	0,00358	**
18:3-PC/di-18:3-DAG (238) 18:2-PC/no-DAG (89)	0,00895	**
18:3-PC/no-DAG (53) 18:1-PC/no-DAG (77)	1,00	
18:3-PC/no-DAG (53) 18:2-PC/no-DAG (89)	1,00	

Full name of PC species. 18:1 PC, sn-1-16:0-sn-2-[14C]18:1 PC; 18:2 PC, sn-1-16:0-sn-2-[14C]18:2 PC; 18:3 PC, sn-1-16:0-sn-2-[14C]18:3 PC

Supplementary Table 5. Endogenous PC and DAG content of microsomal preparations from yeast strain H1246. Standard deviation (SD) in paranthesis (n=3 replicates).

nmol/mg protein		*nmol/PDCT assay*	
DAG	PC	DAG	PC
62 (1,9)	327 (19)	6,2 (0,19)	33 (1,9)

Supplementary Table 6. List of primers used for cloning and expression of *C. sativa* genes in yeast

Gene	orientation	Sequence 5'-3'
DGAT1	fw	TTGGTACCTATGGCTATTTTAGATTCTGGTGGTGGTGGTGTTTCTA CTGCTACTGCTACTGAAAATGGTGGCGGAGAGTTTGTGGATCT
DGAT1	rev	CCGGATCCTCATGACATTGATCCTTTGCG
DGAT2	fw	TTGGTACCTATGGGTGGTTCTAGAGAATTTAGAGCTGATAAATCTT CTGATCAATTTCATTCTACTATTGCTATGGCTATTTGGCTTGGCGCC ATTCACTTCAACA
DGAT2	rev	TTGCGGCCGCTCAAAGAATGTTCAGTTGAAGATCAGG
PDAT	fw	TTGGTACCTATGCCATTAATTCATAGAAAGAAGCCACAAACTCCAC CATCTGAAGAAGTTGTTGCTCCAGATGAGGATTCCCAGAAGAAACC
PDAT	rev	TTGCGGCCGCTCACAGCTTCAGGTCTATTCGG
PDCT	fw	TTGGTACCTATGTCTGCTTCTGCTGCTAAACCAGCTGTTTCTAGAA GACATGTTTCTAATGGTAATAATACTAACAACGTCGCCATTGA
PDCT	rev	TTGCGGCCGCTTAATTGACTAGAGAGTCTTTCGATATCAAAC
PDCT-like	fw	TTGGTACCTATGTCTGATGCTGTTACTAAAACTGTTGTTCCATTAAG AAGAAAATCTAATCCATTAAATGGAAAACACACTAACGGCGTC
PDCT-like	rev	TTGCGGCCGCTTAATCGACTAGGGTGTCTTTCG

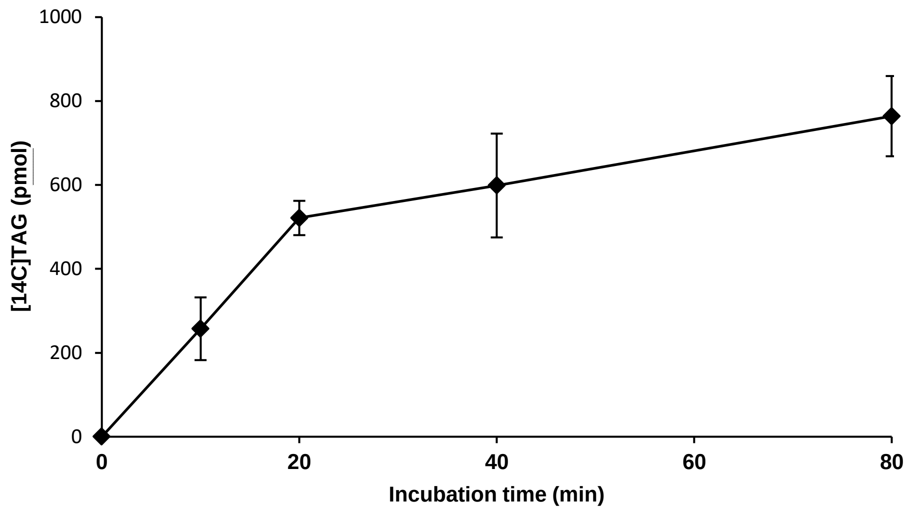

Supplementary Figure 1. Time-course of PDAT activity with exogenous long-chain DAG. Incubations were performed as described in Materials and Method section with [^{14}C]18:2-PC and 18:2-DAG and 60 µg of seed microsomal protein.

frontiers
